# Micelle‐Assisted Encapsulation Strategy in Metal‐Organic Framework Nanocontainers Enables Durable Corrosion Protection

**DOI:** 10.1002/smsc.70367

**Published:** 2026-08-22

**Authors:** Asep Sugih Nugraha, Kwang Keat Leong, Yusuke Yamauchi, Yuan Ma, Nasim Amiralian

**Affiliations:** ^1^ Australian Institute for Bioengineering and Nanotechnology (AIBN) The University of Queensland Brisbane Queensland Australia; ^2^ Baoshan Iron & Steel Co. Ltd. Shanghai China

**Keywords:** corrosion inhibition, metal–organic frameworks, micelle‐assisted encapsulation, self‐healing coatings, waterborne polyurethane

## Abstract

Encapsulation is an effective strategy for stabilizing and controlling functional molecules. Although amphiphilic micelles are widely used to host molecular cargo, their intrinsic encapsulation capability remains underexplored for directly confining functional molecules within inorganic or hybrid architectures. Here, we report a hierarchical nanocontainer integrating micelle‐assisted molecular encapsulation, ZIF‐8 confinement, and SiO_2_ interfacial engineering for smart self‐healing corrosion protection. Amphiphilic P123 micelles first encapsulate benzotriazole (BTA) in situ and direct ZIF‐8 framework growth around the loaded micelles, enabling efficient one‐pot inhibitor confinement without a separate post‐loading step. A subsequent SiO_2_ shell improves structural robustness and compatibility with environmentally friendly waterborne polyurethane (WBPU) while preserving pH‐responsive release. Under corrosive conditions, local pH changes trigger BTA release, enabling active inhibition and self‐healing at damaged sites. The nanocontainers achieve inhibitor loadings up to 16.2 wt% and pH‐dependent release, reaching ~35% at pH 3 and ~32% at pH 10, compared with ~4% at pH 7. Incorporated into WBPU coatings, the composite maintains ~1.4 × 10^7^ Ω·cm^2^ after 39 days in 3.5 wt% NaCl. It also shows a corrosion current density of 3.2 × 10^−9^ A cm^−2^ and a corrosion rate of 0.0000372 mm year^−1^, demonstrating durable protection. This strategy enables high‐loading, release‐regulated nanocontainers for anticorrosion coatings.

## Graphical abstract

A one‐pot strategy integrating micelle‐assisted benzotriazole encapsulation, ZIF‐8 confinement, and SiO_2_ shell formation produces hierarchical nanocontainers with high inhibitor loading and pH‐responsive release. Embedded in waterborne polyurethane coatings, these nanocontainers enable self‐healing corrosion protection, delivering durable long‐term anticorrosion performance through pH‐responsive release.

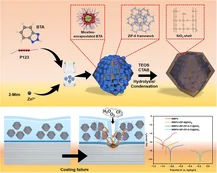

## 1 Introduction

Encapsulation is a powerful and widely adopted strategy for the stabilization and controlled delivery of functional molecules, enabling technologies ranging from drug delivery [1] and catalysis [2] to sensing [3] and energy storage [4, 5]. This strategy is highly relevant to corrosion protection, where molecular inhibitors should remain immobilized within protective carriers during normal service but be released efficiently when corrosion is initiated. Accordingly, capsule‐based systems have been widely developed, in which inhibitors are confined within protective shells that regulate release and isolate the active molecules from the coating matrix until triggered by coating damage or local corrosion processes [6, 7, 8]. Various polymeric shells, including polyurea [9], epoxy [10], polyurethane [11], and poly(urea‐formaldehyde) [12], have been used because they offer flexible capsule design with tunable shell thickness and compatibility with a wide range of inhibitor or healing‐agent molecules. Despite these advantages, capsule‐based systems can still face practical challenges in waterborne coating matrices, particularly dispersion instability, shell fragility, and limited interfacial compatibility with the surrounding polymer matrix.

To address some of these limitations, porous nanocontainers have emerged as promising alternative platforms for active corrosion protection [13]. Their well‐defined and chemically tunable porous structures provide confined spaces for inhibitor storage, helping to reduce premature leaching and rapid depletion while enabling controlled release in response to local corrosion processes [14, 15]. Among these systems, metal‐organic frameworks (MOFs) have attracted substantial attention as functional fillers for organic anticorrosion coatings because their pore structures, metal nodes, and organic linkers can be tailored to regulate inhibitor loading, barrier performance, interfacial interactions, and stimulus‐responsive release [16]. Various MOF platforms, including ZIF‐type [17], UiO‐type [18], MIL‐type [19], and other coordination‐framework‐based nanocontainers [20], have been explored to improve corrosion protection through multiple mechanisms, including physical barrier enhancement [21], inhibitor storage and release [22], pH‐responsive framework degradation [23], metal‐inhibitor complexation [24], and interfacial passivation [25]. In particular, ZIF‐type structures are attractive as practical nanocontainer platforms for anticorrosion coatings because their facile synthesis, good stability under neutral conditions, and pH‐responsive behavior enable efficient preparation and corrosion‐triggered inhibitor release [26]. Accordingly, corrosion inhibitors such as benzotriazole (BTA) have been incorporated into ZIF‐based nanocontainers through pores, defects, interfacial regions, or other framework‐associated confined spaces [27, 28, 29, 30]. However, many ZIF‐based anticorrosion nanocontainers still rely on post‐synthetic inhibitor loading or adsorption, which adds an extra processing step, while one‐step incorporation strategies can still be limited by the range of inhibitor molecules compatible with ZIF formation. This highlights the need for an integrated preparation strategy that combines inhibitor encapsulation with nanocontainer formation to simplify the inhibitor‐loading process, improve inhibitor retention, regulate release behavior, and broaden the molecular versatility of ZIF‐based anticorrosion nanocontainers.

Amphiphilic micelles provide an attractive platform to achieve this integration because they can solubilize and confine molecular cargo within nanoscopic domains [31, 32]. Beyond molecular delivery, micelles are also widely used as soft templates to guide the formation of nanostructures, including hollow and mesoporous architectures [33, 34, 35]. However, in most templating strategies, micelles are used as sacrificial structure‐directing agents and removed after framework formation [36]. Their cargo‐confinement capability remains largely underexplored for directly incorporating functional molecules into nanocontainers. This gap creates an opportunity to use amphiphilic micelles as functional molecular reservoirs that encapsulate BTA while directing ZIF‐based nanocontainer growth. Such micelle‐assisted encapsulation enables inhibitor incorporation during nanocontainer formation, providing a practical route to hierarchical BTA‐loaded ZIF nanocontainers for waterborne anticorrosion coatings.

In addition to the internal storage capability of nanocontainers, surface and interfacial engineering is essential for translating nanocontainers into practical organic coatings. Silica (SiO_2_)‐based shells and hybrid structures are particularly effective because they promote coating integration by stabilizing dispersion, moderating filler–matrix interactions, and improving barrier durability [37, 38, 39]. For ZIF‐based anticorrosion coatings, SiO_2_ encapsulation can further provide a protective interfacial layer that regulates inhibitor release under corrosion‐induced microenvironments [40, 41]. This design offers a promising route to combine active inhibition with improved coating durability. Therefore, we introduce a hierarchical nanocontainer design that integrates micelle‐assisted molecular encapsulation, ZIF‐based confinement, and SiO_2_ interfacial engineering to enhance inhibitor loading and retention while regulating inhibitor release for durable active corrosion protection. In this work, we use amphiphilic P123 micelles that serve a dual function: their micellar cores encapsulate BTA molecules in situ, while their self‐assembled structure directs the growth of a ZIF‐8 framework around the loaded micelles, enabling efficient and stabilized inhibitor confinement. Subsequent coating with a SiO_2_ shell further enhances structural robustness and aqueous stability within the waterborne polyurethane (WBPU) matrix, while preserving stimulus‐responsive release behavior. This integrated architecture enables one‐pot sequential synthesis with a high inhibitor loading of up to 16.2 wt%. The hydrophilic SiO_2_ shell improves compatibility with the WBPU matrix, as indicated by the visually homogeneous suspension and uniform coating morphology. In addition, the hierarchical nanocontainer retains pH‐responsive behavior, enabling pH‐dependent inhibitor release under corrosive conditions. As a result, the composite coating exhibits a low‐frequency impedance modulus |*Z*|_0.01_ Hz of approximately 1.4 × 10^7^ Ω·cm^2^ after prolonged immersion. This micelle‐assisted encapsulation strategy can be extended to other corrosion inhibitors and functional molecules for durable, actively protective coatings.

## 2 Results and Discussion

**FIGURE 1:**
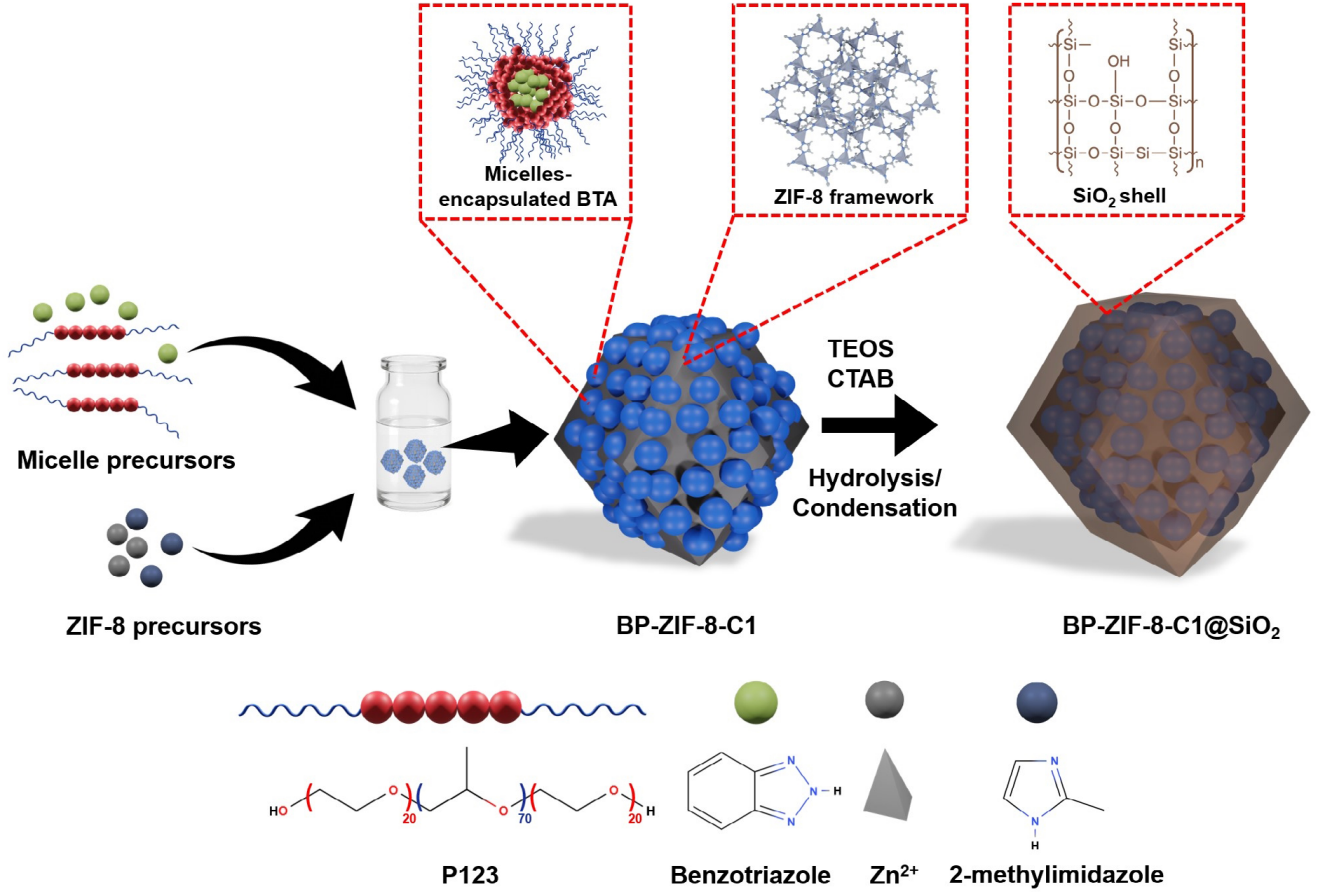
Schematic illustration of the formation of hierarchical BP‐ZIF‐8‐C1@SiO_2_ nanocontainers. P123 micelles encapsulate BTA and guide the initial confined assembly, followed by ZIF‐8 framework growth around the BTA‐loaded micelles. In the final step, TEOS undergoes hydrolysis/condensation to form the outer SiO_2_ shell, with CTAB assisting the controlled deposition of silica around the particles.

**FIGURE 2:**
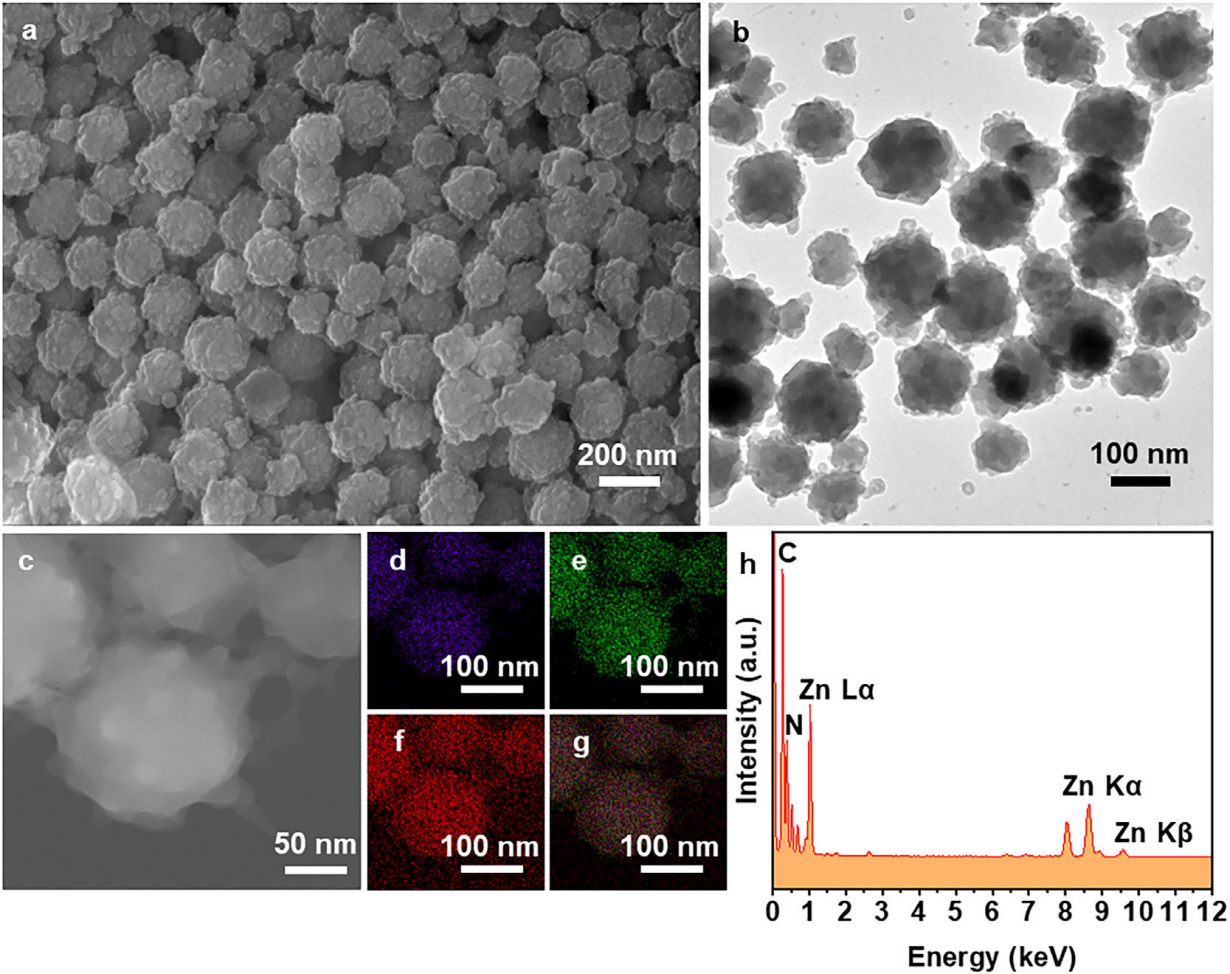
(a) SEM, (b) TEM, and (c) HAADF‐STEM images of BP‐ZIF‐8‐C1. Corresponding STEM‐EDX elemental maps showing the spatial distributions of (d) N, (e) Zn, and (f) C, together with (g) the merged elemental map and (h) the EDX spectrum.

As illustrated in Figure 1, the nanocontainer incorporating BTA was synthesized through a one‐pot sequential approach. The formulation details and corresponding sample nomenclature for the BTA/P123‐assisted ZIF‐8 samples prepared with and without SiO_2_ coating are summarized in Table S1. In the typical synthesis, P123 block‐copolymer micelles are first formed in water, where the amphiphilic PEO–PPO–PEO chains self‐assemble into micelles once the concentration exceeds the critical micelle concentration [42]. The aqueous P123 micellar dispersion is then combined with a THF solution of BTA. Owing to the preferential migration of BTA into the hydrophobic PPO core, driven by its strong affinity for nonaqueous environments and its higher solubility in THF than in water, BTA becomes effectively encapsulated within the micelles. This diffusion‐driven encapsulation is supported by transmission electron microscopy (TEM) analysis, which shows a marked increase in the average micelle diameter upon BTA loading, from 10.4 ± 1.3 nm for P123 micelles (Figure S1a) to 20.9 ± 2.2 nm after BTA incorporation (Figure S1b), with each value calculated from measurements of 200 micelles. This size increase indicates successful encapsulation of BTA within the micellar interior. An aqueous solution containing Zn^2+^ and 2‐MIm is then introduced into the micellar dispersion. Given that the hydrophilic PEO corona may provide interfacial sites capable of binding metal–ligand precursors and initiating localized nucleation, the BTA‐loaded micelles are expected to act as nucleation anchors [43]. In this way, the confined micelles offer localized nucleation sites that promote the growth of the ZIF‐8 framework around the BTA‐encapsulating domains. After 8 h of gentle stirring following the introduction of the ZIF‐8 precursors, the reaction mixture became milky, indicating the formation of colloidally dispersed nanoparticles. The reaction was deliberately terminated at this stage to examine the intermediate structures and assess the successful incorporation of BTA‐loaded micelles within the developing ZIF‐8 framework. For this analysis, the subsequent SiO_2_‐coating step was omitted to allow direct characterization of the ZIF‐8 particles without contributions from the SiO_2_ layer. The obtained ZIF‐8 sample, denoted as BP‐ZIF‐8‐C1, was collected by centrifugation and washed several times with water to remove unreacted species. Scanning electron microscope (SEM) was then conducted to examine the morphology of the as‐synthesized product. As shown in Figure 2a, the particles with an average diameter of 164 ± 16 nm display a bumpy surface texture. This morphology contrasts sharply with the sample prepared in the absence of P123 but in the presence of BTA, denoted as B‐ZIF‐8 (Figure S2a), which exhibits the smooth morphology characteristic of pristine ZIF‐8. The modified surface features suggest the formation of a composite structure in which micelle‐confined BTA is incorporated within the ZIF‐8 framework. TEM images further show that the particles are composed of small, low‐contrast organic‐rich domains enclosed within the structure (Figure 2b). These regions are indicative of the presence of BTA‐loaded micelles embedded inside the ZIF‐8 matrix.

To further verify the presence of micelle‐confined BTA in the as‐synthesized nanoparticles, an additional experiment was conducted in which the sample was thoroughly washed with THF to remove the P123 micelles containing BTA. As shown in the TEM images (Figure S2b), the particles exhibited a distinct porous structure after micelle removal, with an average pore size of 21.6 ± 3.1 nm based on measurements of 200 pores. The pore sizes correspond well to those of the BTA‐loaded micelles seen in TEM (Figure S1b), suggesting that the micelles acted as preferential nucleation sites for ZIF‐8 growth. Interestingly, when BTA was omitted during synthesis, the resulting nanoparticles exhibited a dense, nonporous morphology, with no internal voids (Figure S2c). Previous studies have shown that the incorporation of hydrophobic molecules into Pluronic micelles can enhance micellar stability by reinforcing the hydrophobic PPO core [43, 44]. In line with this observation, the clear contrast between samples prepared with P123 in the presence and absence of BTA suggests that the hydrophobic BTA is encapsulated within the micellar core and plays a key role in stabilizing the P123 micelles. This enhanced stability enables the micelles to maintain a well‐defined core that serves as a localized nucleation domain for ZIF‐8 growth, ultimately generating the internal porosity observed after micelle removal. Scanning transmission electron microscopy (STEM) imaging and energy‐dispersive X‐ray (EDX) analysis further reveal the particle morphology and spatial distribution of elements within the particles (Figure 2c–h). Signals corresponding to Zn, N, and C are observed, indicative of the presence of a ZIF‐8 framework formed around the micelles.

**FIGURE 3:**
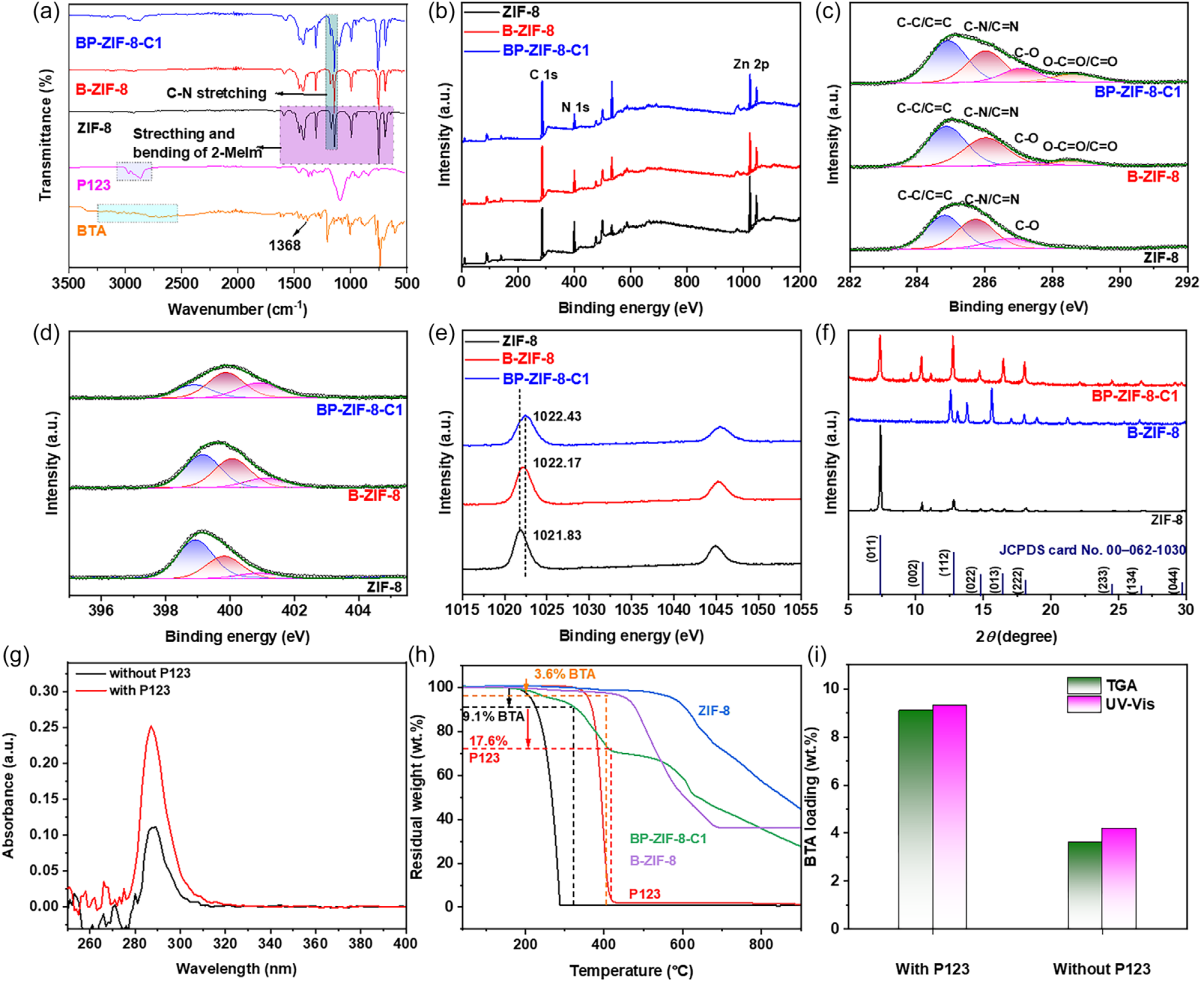
(a) FTIR spectra, (b) wide‐scan XPS spectrum, and high‐resolution XPS spectra of (c) C 1s, (d) N 1s, and (e) Zn 2p. (f) XRD pattern, (g) UV–vis absorption spectra, (h) TGA curves, and (i) the corresponding BTA weight percentages (wt%). The UV–vis measurement was performed by first dissolving the sample in 1 M HCl to prepare a 1 mg mL^−^^1^ stock solution. Then, 600 μL of the stock solution was diluted with 3 mL of water.

A series of characterization studies were carried out to further corroborate the successful encapsulation of BTA and to understand its interaction within the hybrid system. Fourier‐transform infrared (FTIR) analysis was first employed to verify the presence of BTA and P123 in the composites and to examine any changes to the ZIF‐8 framework upon their incorporation. Pristine ZIF‐8 was synthesized as a reference material for comparison (Figure S2d). As shown in Figure 3a, pristine ZIF‐8 exhibits the characteristic vibrational features of the 2‐MIm linker, with multiple bands in the 600–1500 cm^−1^ region corresponding to ring stretching, C—N stretching, and in‐plane/out‐of‐plane bending modes [45, 46]. These defining ZIF‐8 signals remain in both B‐ZIF‐8 and BP‐ZIF‐8‐C1, indicating that the structural integrity of the framework is maintained. Notably, BTA‐containing samples (BP‐ZIF‐8‐C1 and B‐ZIF‐8) show an additional absorption band at ~1368 cm^−1^, which is absent in pristine ZIF‐8. This feature can be assigned to the C—N stretching coupled with ring deformation of BTA and serves as a clear fingerprint confirming the presence of BTA within the composite, even though the broad N–H vibrational envelope of BTA (2500–3400 cm^−1^) cannot be observed [47]. Evidence for P123 incorporation is also apparent in the BP‐ZIF‐8‐C1 spectrum. The appearance of pronounced CH_2_/CH_3_ stretching bands at ~2970 and 2870 cm^−1^, features characteristic of pure P123 and absent in pristine ZIF‐8 or B‐ZIF‐8, provides clear evidence for the incorporation of the triblock copolymer within the composite [48]. Additionally, a subtle increase in the ether C—O—C stretching region near ~1100 cm^−1^ further supports the successful integration of P123 within the hybrid material [49]. X‐ray photoelectron spectroscopy (XPS) analysis was then performed to probe the elemental composition and to understand how P123 affects the chemical environment of the BTA‐loaded samples. Three samples were examined for comparison: pristine ZIF‐8, B‐ZIF‐8, and BP‐ZIF‐8‐C1. As shown in Figure 3b, XPS surveys display characteristic Zn, N, and C signals associated with the ZIF‐8 framework for all samples. Closer inspection of the high‐resolution C 1s spectrum of pristine ZIF‐8 (Figure 3c) reveals three fitted components located at 284.80, 285.73, and 286.73 eV, corresponding to C—C/C—H from the 2‐MIm linker, C—N/C=N associated with nitrogen‐bonded carbon in the imidazolate ring, and a small C—O contribution from surface oxygenated species, respectively [50]. In both BTA‐containing samples, an additional peak appears at ~288.54 eV, which is assigned to high‐binding‐energy carbon species, including possible O—C=O/C=O groups from oxidized surface contaminants and C—N/C=N environments [51]. Its presence in both BTA‐containing samples suggests that the increased surface organic content introduced by BTA facilitates the adsorption of atmospheric CO_2_‐derived carbonate species. The N 1s spectra show that all samples exhibit three peaks corresponding to the coordinated imidazolate nitrogen (Zn—N) at ~398.9 eV, a medium‐binding‐energy contribution at ~399.8 eV attributed to protonated or defect‐associated imidazolate nitrogen (and possible contribution from BTA‐derived N species), and a higher‐binding‐energy component at ~400.9 eV attributed to strongly protonated or positively polarized nitrogen (Figure 3d) [28, 52]. While pristine ZIF‐8 exhibits only minor contributions from the latter two components, both BTA‐containing samples show markedly increased intensities in the ~399–401 eV region, consistent with the incorporation of BTA. Notably, the BP‐ZIF‐8‐C1 sample displays the largest increase, which may be attributed to the differences in the surface distribution or accessibility of BTA‐derived nitrogen species, together with contributions from defect‐associated or protonated imidazolate nitrogen. The decrease in the Zn—N signal suggests attenuation of the ZIF‐8 surface signal by organic components associated with BTA/P123 incorporation. In addition, the Zn 2p spectrum consists of two characteristic peaks corresponding to Zn 2p_3/2_ and Zn 2p_1/2_ [53]. For pristine ZIF‐8, these peaks are located at 1021.88 and 1044.95 eV, respectively (Figure 3e). Upon incorporation of BTA, both Zn 2p_3/2_ and Zn 2p_1/2_ shift slightly toward higher binding energy. This shift indicates a more electron‐deficient Zn environment in the presence of BTA, which may reflect changes in the local Zn—N coordination environment [54, 55]. As BTA may compete with 2‐MIm for interaction with the Zn nodes during synthesis [56], limited Zn—N(BTA) interactions may contribute to the observed positive shift. A further positive shift is observed for BP‐ZIF‐8‐C1 following the introduction of P123, suggesting an additional perturbation of the local Zn environment [54, 55, 56]. In this system, most BTA is confined within the P123 micelles, while BTA molecules located near the micelle/ZIF‐8 interface may remain accessible for localized interaction with Zn species during framework growth. This arrangement enables BTA confinement while preserving the characteristic sod‐ZIF‐8 structure.

X‐ray diffraction (XRD) analysis was conducted to evaluate the preservation of the ZIF‐8 framework. As shown in Figure 3f, pristine ZIF‐8 displays prominent diffraction peaks that align well with the simulated ZIF‐8 pattern, with characteristic reflections at 2*θ* = 7.2°, 10.3°, 12.7°, 14.7°, 16.4°, 18.0°, 24.5°, 26.7°, and 29.6°, corresponding to the (011), (002), (112), (022), (013), (222), (233), (134), and (044) planes, respectively (JCPDS 00‐062‐1030) [57]. The diffraction pattern of BP‐ZIF‐8‐C1 closely matches that of pristine ZIF‐8, indicating that the presence of P123 micelles preserves the sod‐type crystallization pathway without disrupting framework crystallinity. In contrast, B‐ZIF‐8 prepared in the absence of micellar confinement exhibits noticeable differences in peak positions relative to pristine ZIF‐8, suggesting the formation of a different, denser Zn(mIm)_2_ polymorph [58]. These observations suggest that strong interactions between BTA molecules and Zn^2+^ ions in aqueous media can perturb the conventional Zn–2‐MIm self‐assembly process, thereby favoring the formation of a denser phase [59, 60]. Notably, the introduction of P123 micelles acts as a physical and chemical barrier that spatially isolates BTA molecules from direct coordination with Zn^2+^, thereby suppressing premature phase diversion and allowing Zn–2‐MIm assembly to proceed toward sod‐ZIF‐8 formation. This crystallographic behavior is consistent with the altered local Zn environment suggested by the XPS analysis, which may involve localized Zn—N(BTA) interactions. The XRD observations also agree well with the electron microscopy data (Figure 2a), further validating the micelle‐assisted encapsulation strategy.

Quantitative analysis was carried out to determine the amount of BTA incorporated into the as‐prepared samples, an essential parameter for evaluating the performance of the nanocontainer system. UV–vis spectroscopy was first employed for this purpose. The samples were dissolved in 1 M HCl to fully decompose the ZIF‐8 framework and release all BTA. As shown in Figure 3g, all samples exhibit a characteristic absorption peak at 287 nm, which corresponds to the π→π* transition of the BTA aromatic ring [61]. Quantitative analysis based on the calibration curve (Figure S3) indicates that the B‐ZIF‐8 sample contains approximately 4.2 wt% BTA, whereas the BP‐ZIF‐8‐C1 sample shows a substantially higher loading of about 9.3 wt%. This enhancement indicates that the presence of P123 micelles improves BTA incorporation, likely by stabilizing hydrophobic BTA within the micellar core during ZIF‐8 crystallization. The BTA loading of the samples was also evaluated using Thermogravimetric analysis (TGA). Figure 3h illustrates that the TG curve of BP‐ZIF‐8‐C1 features three major weight‐loss stages. The initial mass loss below ~220 °C corresponds to the release of adsorbed and physically entrapped water within the structure. The second, sharp weight‐loss step between 220 and 320 °C is assigned to the thermal decomposition of BTA, consistent with its characteristic decomposition temperature observed in the TG profile. After BTA decomposition, a third weight‐loss event occurs due to the thermal degradation of the P123 surfactant confined within the ZIF‐8 framework. At temperatures above ~530 °C, the final decrease in mass corresponds to the decomposition of the ZIF‐8 organic linker, leading to collapse of the framework. From the mass loss in the BTA decomposition region (220–320 °C), BP‐ZIF‐8‐C1 is calculated to contain ~9.1 wt% BTA, whereas B–ZIF‐8 contains only ~3.6 wt%. These results align well with the UV–vis quantification, confirming that the incorporation of P123 micelles significantly enhances BTA loading (Figure 3i). Additionally, the thermal stability of the samples provides further insight into the role of P123 encapsulation. B‐ZIF‐8 shows a clear reduction in thermal stability, with the main decomposition event occurring at approximately 480 °C. This behavior indicates perturbation of the ZIF framework and is attributed to the formation of a denser Zn(mIm)_2_‐type phase rather than sod‐ZIF‐8. Such dense ZIF polymorphs are known to exhibit distinct thermal decomposition behavior compared to sod‐type ZIF‐8 [59]. In contrast, the BP‐ZIF‐8‐C1 sample exhibits a thermal decomposition profile nearly identical to that of pristine ZIF‐8, with the primary framework degradation occurring at around 550 °C. This preserved thermal stability indicates that the introduction of P123 micelles effectively regulates the crystallization pathway, enabling the formation of phase‐pure sod‐ZIF‐8.

Compared with previously reported molecular adsorption, hydrogen‐bond‐assisted loading, and linker‐substitution strategies [62, 63, 64], the present P123‐assisted approach is designed to regulate the local confinement of BTA during ZIF‐8 formation. To clarify the role of the BTA/P123 formulation in this process, samples with different precursor compositions were investigated. As shown in the TEM images in Figure S4, the particle morphology was strongly affected by increasing the BTA amount while keeping the P123 concentration constant. For BP‐ZIF‐8‐R1.19, where the BTA amount was increased to 2.5 times the initial amount, the particles became smaller, with an average size of approximately 20 nm, and exhibited visible hollow cavities. Further increasing the BTA amount to 5 times the initial concentration in BP‐ZIF‐8‐R2.38 led to even smaller particles with more pronounced hollow features. These formulations correspond to BTA/P123 mass ratios of 1.19 and 2.38, respectively. This behavior suggests that the excess BTA precursor, which exceeds the encapsulation capacity of P123 micelles, remains unconfined and can partially replace or interfere with the 2‐MIm linkers during crystallization. Such disruption of the coordination environment likely prevents ZIF‐8 from fully capping the BTA‐loaded micelles, resulting in structural defects and incomplete shell formation. In contrast, when the amounts of both BTA and P123 were proportionally increased to 2.5 times their initial concentrations, the resulting sample, denoted as BP‐ZIF‐8‐C2, largely retained the original particle morphology, as further confirmed by STEM‐EDX analysis (Figure S5a–g). This consistency indicates that maintaining an appropriate BTA/P123 ratio is crucial for effective micelle encapsulation and for sustaining the structural integrity of the resulting composite. Notably, increasing both the BTA and P123 amounts by 2.5‐fold while maintaining a constant BTA/P123 mass ratio of 0.48 increased the BTA loading to 16.2 wt% (Figure S5h), consistent with the greater availability of BTA for micelle‐assisted encapsulation. These morphological observations are further supported by XRD analysis (Figure S6). In samples containing elevated BTA levels but insufficient P123 (BP‐ZIF‐8‐R1.19 and BP‐ZIF‐8‐R2.38), the diffraction peaks remain at positions consistent with simulated ZIF‐8; however, their significantly reduced intensity and pronounced broadening indicate diminished crystallinity. In contrast, BP‐ZIF‐8‐C2 prepared with a balanced BTA/P123 ratio displays sharp and well‐defined diffraction peaks, implying that effective micelle encapsulation is critical for preserving the crystalline ZIF‐8 framework. Collectively, these results demonstrate that an appropriately balanced BTA/P123 ratio is essential for maximizing BTA loading while preserving the morphological stability and crystalline quality of the micelle‐assisted ZIF‐8 nanocontainers. Moreover, these findings demonstrate that optimizing the BTA/P123 ratio not only preserves the morphology and crystallinity of the micelle‐assisted ZIF‐8 nanocontainers but also enables a high BTA loading comparable to previously reported anticorrosion nanocontainer systems, as summarized in Table S2. Importantly, this micelle‐assisted encapsulation strategy is not limited to BTA and may be extended to other small organic corrosion inhibitors with compatible solubility and micelle‐partitioning behavior. In this system, micelle encapsulation of corrosion inhibitor molecules is feasible for molecules that exhibit good solubility and compatibility with an organic cosolvent, such as THF; can partition into the PPO‐rich core or PEO/PPO interfacial region of P123 micelles during micelle assembly; have molecular dimensions suitable for confinement within micelle‐derived nanodomains; remain stable during ZIF‐8 growth and SiO_2_ coating; and retain corrosion‐active donor sites, such as N‐, S‐, or O‐containing groups. As a proof of concept, BTA was successfully substituted with 2‐mercaptobenzothiazole (MBT) in this system (Figure S7), demonstrating the adaptability of the approach toward structurally related heterocyclic inhibitors.

To achieve smart self‐healing anticorrosion behavior, corrosion‐inhibitor nanocontainers must exhibit well‐defined stimulus‐responsive characteristics, such as pH sensitivity arising from local environmental changes during corrosion initiation [65]. While ZIF‐8 undergoes framework degradation under acidic conditions, enabling inhibitor release, an additional SiO_2_ shell was incorporated to introduce complementary pH‐responsive behavior under basic conditions and to enhance the overall structural robustness of the nanocontainers. In this strategy, the SiO_2_ coating step was sequentially introduced after ZIF‐8 formation within the same one‐pot sequential synthesis, taking advantage of the ability of SiO_2_ to grow conformally on preformed ZIF‐8 nanocontainers during the synthesis. As shown in Figure S8a,b, the resulting BP‐ZIF‐8‐C1@SiO_2_ particles exhibit an increased average particle size of approximately 203 ± 11 nm, attributable to the formation of the SiO_2_ shell, while maintaining the overall particle morphology. STEM‐EDX analysis further confirms the successful formation of a conformal SiO_2_ shell with an estimated thickness of 20 ± 3 nm surrounding the ZIF‐8 core (Figure S8c–i). The presence of the SiO_2_ layer is also verified by XPS (Figure S8j), which shows characteristic Si signals on the particle surface. Importantly, XRD patterns of the BP‐ZIF‐8‐C1@SiO_2_ sample reveal that the crystalline structure of ZIF‐8 is preserved after SiO_2_ coating, indicating that the shell formation does not disrupt the underlying framework (Figure S8k).

**FIGURE 4:**
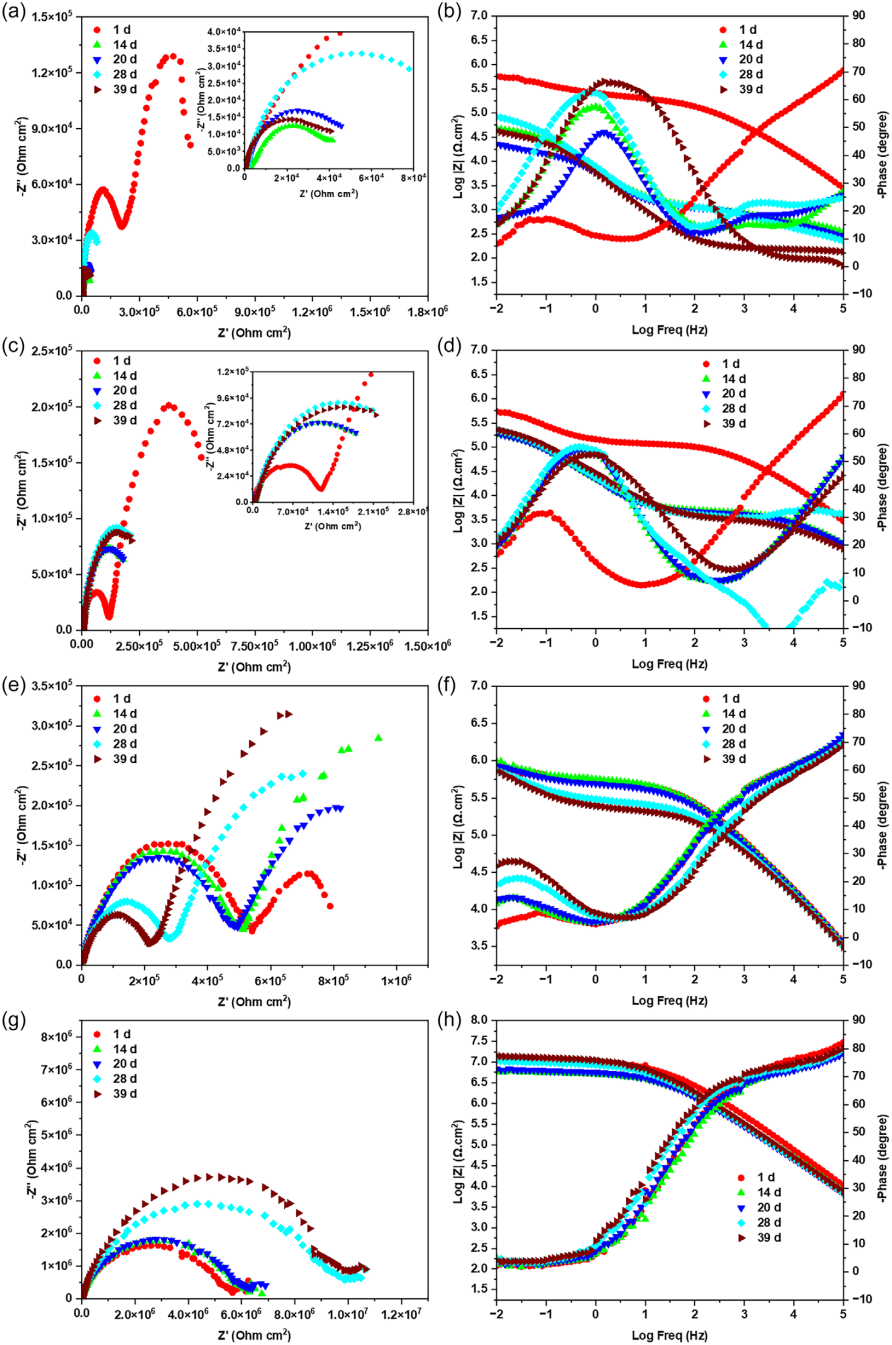
Nyquist plots (a,c,e,g) and corresponding impedance modulus and phase‐angle plots (b,d,f,h) of coated substrates: WBPU (a,b), WBPU + ZIF‐8@SiO_2_ (c,d), WBPU + BP‐ZIF‐8‐C1@SiO_2_ (e,f), and WBPU + BP‐ZIF‐8‐C2@SiO_2_ (g,h).

The anticorrosion performance of the active corrosion‐inhibiting nanocontainers was assessed by preparing a series of WBPU‐based coatings and evaluating them through quantitative and qualitative analyses. Pristine WBPU was used as the polymer matrix reference, while WBPU containing ZIF‐8@SiO_2_ served as an inhibitor‐free nanocontainer control (Figure S9). This control was included to distinguish the intrinsic barrier/filler contribution of the nanocontainer structure from the active inhibition effect provided by BTA loading and release. To examine the influence of inhibitor loading, coatings containing BP‐ZIF‐8‐C1@SiO_2_ and BP‐ZIF‐8‐C2@SiO_2_ were prepared. BP‐ZIF‐8‐C2@SiO_2_ was synthesized using 2.5‐fold higher amounts of both BTA and P123 precursors, as characterized in Figure S10. For all formulations, the nanocontainers were added at a concentration of 1 wt% relative to the WBPU matrix and dispersed under gentle stirring until a homogeneous suspension was obtained. As shown in Figure S11, the coating suspension after stirring appeared visually homogeneous, with no obvious aggregation or phase separation. This indicates that the nanocontainers were reasonably dispersed in the WBPU matrix after stirring. The coating matrix was deposited onto cleaned metal plates using a bar coater and then cured at 50 °C for 12 h to obtain solid coating films. After curing, the dry coating thickness was measured at multiple positions across each coated substrate. The average coating thicknesses are provided in Table S3, showing that all coating systems had comparable thicknesses with mean values in the range of approximately 59–66 μm. As shown in Figure S12, optical microscopy analysis reveals uniform surface topography for all coatings, with no observable macroscopic defects even in the presence of nanocontainers. Cross‐sectional SEM images of a typical coating further show a continuous coating layer  without obvious nanocontainer aggregation or phase separation within the WBPU matrix (Figure S13). Elemental mapping also shows broadly distributed constituent elements across the coating area across the coating area (Figure S14). The corrosion protection performance of WBPU coatings incorporating different nanocontainers was evaluated by electrochemical impedance spectroscopy (EIS) during immersion in 3.5 wt% NaCl solution over different immersion times, as EIS provides a sensitive probe of coating barrier properties. To gain mechanistic insight into the barrier performance of the coatings, the EIS spectra were first analyzed using Nyquist plots. In Nyquist plots, a larger high‐frequency semicircular arc indicates superior barrier performance of the coating by providing higher resistance to electrolyte penetration [66]. In coated systems, this capacitive response is mainly governed by the coating layer, while charge–transfer processes at the metal/coating interface typically appear at lower frequencies. As shown in Figure 4a,c,e,g, after 39 days of immersion, incorporation of ZIF‐8@SiO_2_ into the WBPU matrix results in a noticeably enlarged capacitive loop compared with pristine WBPU coating, indicating enhanced barrier properties even in the absence of corrosion inhibitors. This improvement may be attributed to the combined contributions of uniformly dispersed rigid ZIF‐8@SiO_2_ nanocontainers, which increase coating tortuosity and disrupt continuous ion‐transport pathways, and the possible active protection associated with partial ZIF‐8 decomposition, where framework‐derived species can adsorb on active corrosion sites at the metal/coating interface [67, 68]. In addition, the SiO_2_ shell improves interfacial compatibility between the inorganic nanocontainers and the WBPU matrix, reducing microvoids and defects that typically serve as preferential pathways for electrolyte ingress. Upon incorporation of BTA, the WBPU coating containing the BP‐ZIF‐8‐C1@SiO_2_ system exhibits a substantially larger semicircle than both pristine WBPU and ZIF‐8@SiO_2_‐filled coatings, demonstrating a further enhancement in corrosion protection arising from the combined barrier and inhibitor effects. Notably, the higher BTA loading achieved in the BP‐ZIF‐8‐C2@SiO_2_ coating leads to the most pronounced improvement in barrier performance, as evidenced by the largest capacitive loop among all samples. To further corroborate the Nyquist analysis, the corresponding impedance modulus and phase‐angle plots are shown in Figure 4b,d,f,h. In general, a higher low‐frequency impedance modulus at 0.01 Hz (|Z|_0.01_ Hz) directly reflects an enhanced ability of the coating to block electrolyte penetration and suppress interfacial charge transfer. Meanwhile, the evolution of the phase angle provides complementary insight into coating integrity through the presence and separation of distinct time constants, with high‐frequency responses originating from the coating matrix and low‐frequency features indicating electrolyte access to the coating/metal interface. As shown in Figure 4b, pristine WBPU exhibits an initially high |Z|_0.01_ Hz (~5.9 × 10^5^ Ω·cm^2^) after 1 day of immersion, consistent with an intact polymer barrier. However, rapid degradation occurs upon prolonged exposure, as evidenced by a sharp decrease in |*Z*|_0.01_ Hz to ~4.6 × 10^4^ Ω·cm^2^ and a collapse of the high‐frequency phase angle from ~70° to ~25°, indicating severe loss of dielectric behavior and accelerated ionic transport through the coating. The simultaneous emergence of a pronounced low‐frequency phase response signifies early activation of interfacial corrosion processes, indicating that the long‐term protective capability is limited. In contrast, WBPU composite coatings incorporating ZIF‐8@SiO_2_ nanocontainers display markedly improved barrier stability. Although |Z|_0.01_ Hz decreases moderately over time, it remains at ~2.3 × 10^5^ Ω·cm^2^ even after 39 days of immersion, accompanied by a sustained high‐frequency phase angle of ~50°. This behavior suggests that the nanocontainers increase ion‐transport tortuosity and preserve the capacitive response of the coating, while partial decomposition of ZIF‐8 may also contribute to active protection through the release of framework‐derived species that adsorb on active corrosion sites at the metal/coating interface, as previously reported [68]. A further enhancement in coating barrier performance is achieved by incorporating encapsulated BTA within the nanocontainers. The WBPU coating containing BP‐ZIF‐8‐C1@SiO_2_ exhibits a high initial |Z|_0.01_ Hz (~8.9 × 10^5^ Ω·cm^2^) that remains essentially unchanged after 39 days, together with a stable high‐frequency phase maximum of ~70° and only a weak low‐frequency response. Increasing the BTA loading in the BP‐ZIF‐8‐C2@SiO_2_ system results in an exceptional impedance level of ~1.4 × 10^7^ Ω·cm^2^, with the phase‐angle response dominated by a single time constant (~80°). These results suggest a synergistic protection mechanism in which the enhanced physical barrier provided by the uniformly dispersed ZIF‐8@SiO_2_ nanocontainers is complemented by active interfacial inhibition from the pH‐dependent release of BTA, together mitigating charge–transfer processes and delaying the onset of corrosion [69, 70]. Furthermore, the EIS spectra were quantitatively analyzed by fitting them to the proposed equivalent circuit model (Figure S15), allowing the contributions of the coating barrier and the metal/coating interface to be decoupled. The fitting results (Table S4) show that the evolution of both the coating resistance (*R*_c_) and the charge–transfer resistance (*R*_ct_) is strongly dependent on the coating formulation. While the addition of ZIF‐8@SiO_2_ into the WBPU coatings exhibits only limited increases in *R*_c_ and relatively low *R*_ct_ values upon prolonged immersion, coatings containing BTA‐loaded nanocontainers display a pronounced and sustained enhancement in both parameters. In particular, the high‐loading BP‐ZIF‐8‐C2@SiO_2_ coating exhibited the highest *R*_c_ and *R*_ct_ values after 39 days, reaching 1.26 × 10^7^ and 2.66 × 10^6^ Ω⋅cm^2^, respectively. The BP‐ZIF‐8‐C1@SiO_2_ coating also showed a marked increase in *R*_ct_, indicating enhanced interfacial corrosion resistance. These results suggest that the BTA‐containing nanocontainers contributed to sustained long‐term protection, with the higher‐loading system providing the strongest overall barrier and interfacial resistance. While EIS primarily reflects the barrier properties of the coating, potentiodynamic polarization measurements were performed to assess corrosion kinetics at the coating/metal interface after 39 days of immersion. The corrosion potential (*E*_corr_) and corrosion current density (*i*_corr_) were extracted via Tafel extrapolation, where a lower *i*_corr_ indicates a lower estimated corrosion rate, whereas shifts in *E*_corr_ reflect changes in the relative anodic and cathodic processes [71]. As can be seen in Figure S16 and Table S5, consistent with the EIS results, the WBPU coating containing ZIF‐8@SiO_2_ exhibits a significantly lower *i*_corr_ (9.87 × 10^−8^ A·cm^−2^) than pristine WBPU (2.08 × 10^−6^ A·cm^−2^), confirming effective suppression of corrosion reactions. Incorporation of encapsulated BTA further enhances inhibition, yielding a more positive *E*_corr_ (−0.823 V) and a reduced *i*_corr_ (3.58 × 10^−8^ A·cm^−2^). Notably, increasing the BTA loading results in a pronounced shift of *E*_corr_ to −0.173 V and an ultralow *i*_corr_ of 3.2 × 10^−9^ A·cm^−2^, highlighting the combined effect of a persistent barrier and sustained interfacial inhibition on long‐term corrosion protection.

**FIGURE 5:**
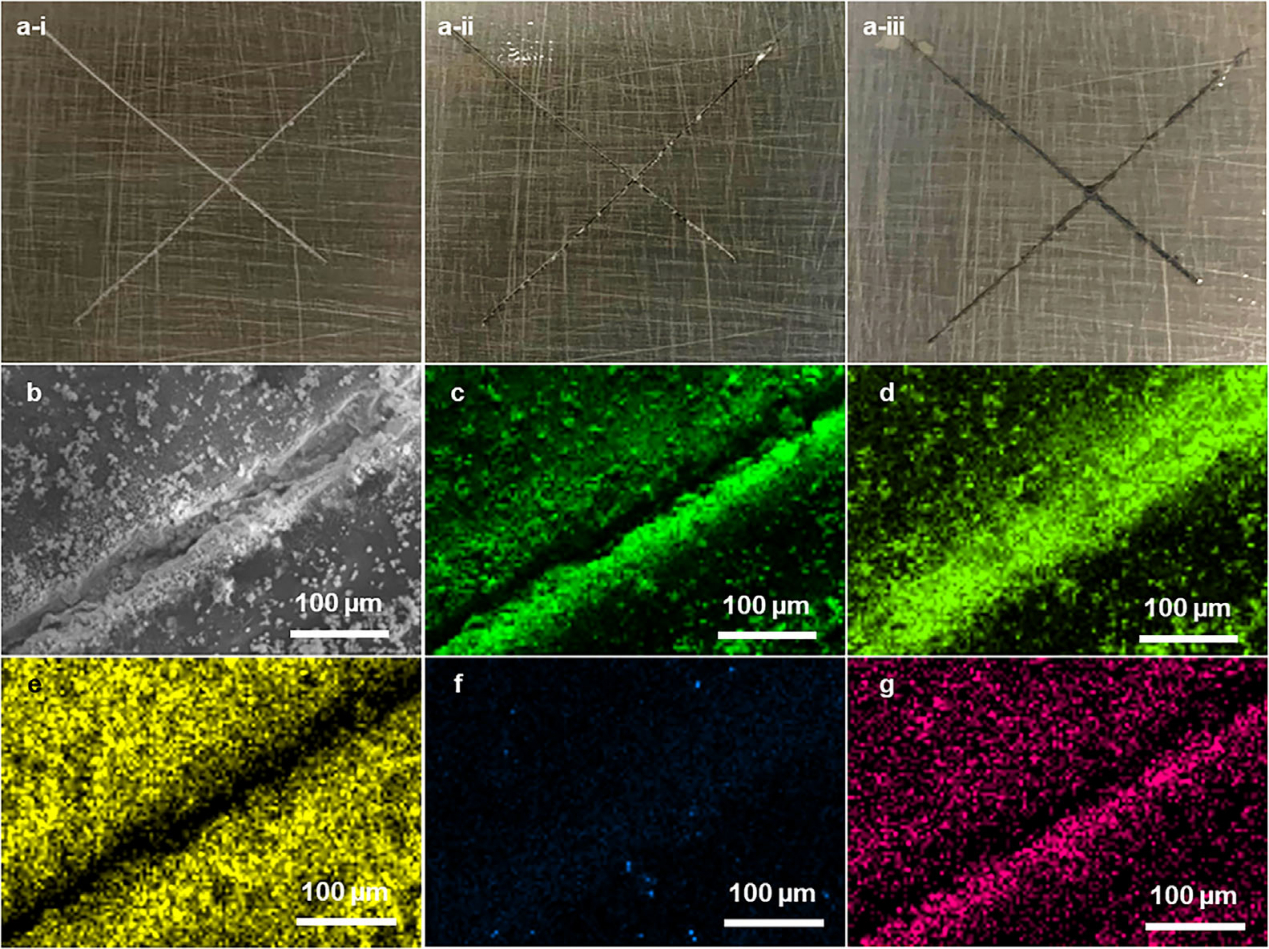
(a) Optical image of the WBPU + BP‐ZIF‐8‐C2@SiO_2_ coating after immersion in 3.5 wt% NaCl for (i) 0, (ii) 7, and (iii) 14 days. (b) SEM image of the scratched region of WBPU + BP‐ZIF‐8‐C2@SiO_2_ coating after 14 days. The corresponding EDX elemental maps show the spatial distributions of (c) O, (d) Zn, (e) Si, (f) Fe, and (g) N.

To examine the robustness of this protection under conditions where the barrier is locally compromised, 14‐day immersion tests were then performed in 3.5 wt% NaCl solution using an intentional surface cut to expose the underlying metal substrate. This defect‐assisted configuration enables direct evaluation of corrosion resistance under sustained and aggressive environmental exposure. In this study, the BP‐ZIF‐8‐C2@SiO_2_ coating was selected as a representative inhibitor‐loaded nanocontainer system to clearly highlight the contrast in protection behavior due to the highest BTA loading. As can be seen in Figure S17a, the uncoated steel exhibits visible signs of corrosion after 7 days, marked by the onset of surface discoloration consistent with chloride‐induced corrosion in the NaCl environment. After 14 days of immersion, extensive reddish‐brown corrosion products are observed, indicating severe corrosion and confirming the high susceptibility of steel under saline conditions. In contrast, all coated samples maintain an effective barrier after 7 days of immersion, with no visible corrosion features. For the WBPU coating, however, degradation becomes evident after 14 days (Figure S17b), as a visibly degraded or discolored region is observed in the vicinity of the artificial defect. This behavior may be attributed to the presence of hydrophilic functional groups within the WBPU matrix, potentially promoting water uptake and contributing to the formation of internal microvoids during drying. These defects can facilitate electrolyte diffusion and lead to local coating delamination from the substrate, highlighting the intrinsic limitation of pristine WBPU for prolonged corrosion protection. For the WBPU coating incorporating ZIF‐8@SiO_2_, no pronounced surface discoloration is observed compared with pristine WBPU, indicating improved corrosion resistance (Figure S17c). Although minor corrosion features appear locally near the artificial defect after 14 days of immersion, their extent is markedly reduced. This behavior suggests that the incorporation of ZIF‐8@SiO_2_ nanocontainers can enhance the micro/nanoscale packing density within the coating matrix, thereby increasing barrier integrity and hindering direct penetration of the corrosive medium. As a result, electrolyte diffusion through the coating is significantly suppressed, and the overall protective performance exceeds that of pristine WBPU. Nevertheless, the emergence of localized corrosion at the cut indicates that, in the absence of an active inhibitor, the exposed metal surface remains vulnerable once direct contact with the saline solution occurs. By comparison, the WBPU coating incorporating BP‐ZIF‐8‐C2@SiO_2_ (Figure 5a) exhibits exceptional resistance to corrosion even in the presence of an artificial defect. After 14 days of immersion, no discernible surface discoloration or corrosion products are observed, including at the cut site, indicating effective suppression of localized corrosion. This enhanced performance can be attributed to the dual protection mechanism enabled by the hierarchical nanocontainers. Under neutral conditions, the ZIF‐8@SiO_2_ framework remains sufficiently stable, allowing the nanocontainers to reinforce the coating barrier and restrict electrolyte ingress. When corrosion is initiated at the artificial defect, local pH changes occur as a result of anodic metal dissolution and cathodic oxygen reduction [72]. These acidic/alkaline microenvironments act as the stimulus for BTA release. Under acidic conditions, protonation of the imidazolate linkers weakens the Zn—N coordination within ZIF‐8, partially opening the framework and facilitating BTA diffusion from the P123/ZIF‐8 domain [73]. Under alkaline conditions, pH‐dependent changes in the SiO_2_ shell, together with possible destabilization of Zn‐based coordination sites, can further increase diffusion pathways through the outer shell [74, 75]. Therefore, BTA release is proposed to arise from the combined effects of pH‐responsive ZIF‐8 destabilization, diffusion through the SiO_2_ layer, and pH‐dependent changes in the silica shell. The released BTA can then adsorb onto the exposed metal surface and form inhibitory interfacial species, suppressing localized corrosion at the damaged site. This proposed pH‐triggered release mechanism is supported by UV–vis release studies, which show pronounced BTA release under acidic and alkaline conditions (~35% at pH 3 and ~32% at pH 10), in contrast to minimal release (~4%) under pH 7 (Figure S18). Consequently, pH‐triggered, on‐demand inhibitor release effectively compensates for local barrier disruption and suppresses the initiation and propagation of corrosion under sustained saline exposure. SEM–EDX analysis of the scratched region of the WBPU coating incorporating BP‐ZIF‐8‐C2@SiO_2_ after immersion (Figure 5b–g) provides spatial evidence supporting the proposed protection mechanism. No obvious accumulation of corrosion products was observed at the defect, while the Zn and N signals were distributed across the exposed region. The N signal indicates the presence of nitrogen‐containing species or nanocontainer components near the damaged area, which may be associated with the transport of BTA‐derived inhibitory species toward the metal surface. The superior performance of the BP‐ZIF‐8‐C2@SiO_2_‐containing coating highlights the synergy between barrier reinforcement and dynamically triggered interfacial inhibition enabled by micelle‐assisted nanoconfinement. The complementary pH responsiveness of ZIF‐8 and SiO_2_ shell may facilitate pH‐dependent BTA release in response to corrosion‐induced local pH fluctuations at defect sites. This design ensures targeted inhibitor delivery where the barrier is compromised, offering a general strategy for encapsulating and preserving inhibitor molecules via micelle‐assisted nanoconfinement in durable, actively protective coatings.

## 3 Conclusion

In summary, we report a micelle‐assisted strategy for loading the corrosion inhibitor BTA by exploiting the intrinsic encapsulation capability of P123 micelles in a one‐pot sequential synthesis. In this approach, BTA molecules are confined within micelles embedded in an integrated micelle–ZIF‐8–SiO_2_ architecture, in which inhibitor storage and release are intrinsically coupled to the hierarchical structure of the nanocontainer. P123 plays a dual role by encapsulating BTA within its micellar core and simultaneously directing the growth of a ZIF‐8 framework around the loaded micelles, enabling spatially confined and stabilized inhibitor loading with enhanced capacity. Subsequent deposition of a SiO_2_ shell further reinforces the hybrid architecture and improves compatibility with the WBPU matrix while preserving accessibility for stimulus‐responsive release. As a result, this efficient loading strategy leads to a pronounced enhancement in anticorrosion coating performance. Beyond BTA, this micelle‐assisted hierarchical encapsulation approach may be extended to compatible small hydrophobic inhibitors, establishing a potentially adaptable strategy for durable, actively protective coatings based on nanoconfined species.

## 4 Experimental Section

### 4.1 Materials and Chemicals

Zinc nitrate hexahydrate (Zn(NO_3_)_2_·6H_2_O, 99%) was purchased from FUJIFILM Wako Pure Chemical Corporation. Pluronic P123 (EO_20_–PO_70_–EO_20_) and 2‐mercaptobenzothiazole (MBT) were obtained from Sigma–Aldrich. 2‐methylimidazole (2‐MIm) (98%), 1H‐benzotriazole (BTA, 99%), tetraethyl orthosilicate (TEOS, 98%), cetyltrimethylammonium bromide (CTAB, 98%), sodium hydroxide (NaOH, 95%), ammonia solution 25 wt%, and tetrahydrofuran (THF, ≥99%) were purchased from Merck. Hydrochloric acid (HCl, 34–37 wt% in H_2_O) was obtained from Thermo Fisher. Sodium chloride (NaCl) was obtained from Chem‐Supply. All chemicals were used as received without further purification. WBPU resin (PU‐8099A, an anionic aliphatic WBPU resin with a solid content of 35 ± 1%, pH of 7–9, and viscosity of ≤300 mPa·s at 25 °C) was obtained from Shanghai Polymer New Material Technology. Zinc‐coated steel plates were supplied by Baosteel Pte. Ltd.

### 4.2 Characterization

A field‐emission scanning electron microscope (FESEM, JEOL JSM‐7100F) operating at 15 kV was used to examine the morphology of the nanocontainers. Transmission electron microscopy (TEM, Hitachi HF5000) equipped with an EDX detector was employed to obtain TEM, high‐resolution TEM (HRTEM), high‐angle annular dark‐field scanning TEM (HAADF‐STEM), and elemental mapping images of the samples. Additional elemental analysis and X‐ray mapping of the coatings were performed using a HITACHI SU3500‐A SEM fitted with an Oxford X‐Max SDD EDS detector. FTIR analysis was carried out using a Thermo Nicolet 5700 spectrometer equipped with a diamond attenuated total reflectance (ATR) accessory to investigate the samples in the wavenumber range of 400–3500 cm^−1^, using a resolution of 4 cm^−1^ and an average of 256 scans. The coating was characterized using a DSX1000 optical microscope to assess its surface morphology and roughness. UV–vis absorption spectra were measured using a Cary 4000 UV–vis spectrometer. Powder XRD measurements were carried out using a Bruker D8 Advance diffractometer with Cu Kα radiation, operating at a scanning rate of 0.2° min^−1^ to examine the crystallinity of the samples. XPS was performed using a Kratos Axis Ultra instrument equipped with a monochromatic Al Kα source (1486.69 eV). All binding energies were calibrated to the C 1s peak at 285 eV. TGA was conducted using a Mettler Toledo TGA/DSC1 Star System. The coating thickness was measured using an Elcometer 456 Coating Thickness Gauge.

### 4.3 Preparation of BP‐ZIF‐8‐C1

BP‐ZIF‐8‐C1 was synthesized by first preparing a 2 M BTA stock solution by dissolving 119 mg of BTA in 500 μL of THF (solution A). Separately, a Pluronic P123 micellar solution was obtained by dissolving 250 mg of P123 in 20 mL of deionized water (solution B). Solution A was added to solution B, followed by sonication and magnetic stirring for 5 min. Subsequently, 1940 mg of 2‐MIm dissolved in 200 mL of deionized water was introduced under gentle stirring. After 30 min, 1190 mg of Zn(NO_3_)_2_·6H_2_O dissolved in 200 mL was added to initiate ZIF‐8 formation, and the reaction was allowed to proceed for 8 h. The resulting solid was collected by centrifugation, washed twice with deionized water, and freeze‐dried.

BP‐ZIF‐8‐C2 was prepared using the same procedure, except that the amounts of BTA and P123 were increased to 2.5 times those used for BP‐ZIF‐8‐C1. Specifically, 297.5 mg of BTA was dissolved in 500 μL of THF, and 625 mg of P123 was dissolved in 20 mL of deionized water, while the amounts of 2‐MIm and Zn(NO_3_)_2_·6H_2_O were kept unchanged. BP‐ZIF‐8‐R1.19 and BP‐ZIF‐8‐R2.38 were synthesized following the same procedure, using different amounts of BTA and P123 as specified in Table S1. For comparison, B‐ZIF‐8 and pristine ZIF‐8 were synthesized following the same procedure, except that Pluronic P123 was omitted for B‐ZIF‐8, whereas both BTA and Pluronic P123 were omitted for pristine ZIF‐8. MP‐ZIF‐8 was synthesized using the same procedure as BP‐ZIF‐8‐C1, with BTA replaced by MBT at the same molar concentration.

### 4.4 Preparation of BP‐ZIF‐8‐C1@SiO_2_

The synthesis followed the same procedure described above for BP‐ZIF‐8‐C1 until the addition of Zn(NO_3_)_2_·6H_2_O, after which the reaction mixture was left to proceed for 8 h. A solution of CTAB (625 mg in 200 mL of water) was then added, followed by 2 mL of ammonia (25 wt%). Finally, 5 mL of a 25 wt.% TEOS solution in water was introduced to form the SiO_2_ shell. The reaction was continued for an additional 3 h. The resulting core–shell product was collected by centrifugation, washed thoroughly with water, and dried using a freeze dryer. For comparison, BP‐ZIF‐8‐C2@SiO_2_ and ZIF‐8@SiO_2_ were prepared following the same procedure.

### 4.5 BTA Loading Measurements

The BTA loading was determined using two methods: TGA and UV–vis spectroscopy. For the TGA‐based analysis, the BTA loading was estimated from the additional weight loss of BP‐ZIF‐8‐C1 compared with pristine ZIF‐8 within the characteristic decomposition region of pure BTA. The TGA‐derived BTA loading was calculated using Equation (1):

(1) BTA loading (wt.%)=ΔW(BP-ZIF-8-C1, BTA region)−ΔW(ZIF-8, BTA region)

where Δ*W* represents the weight loss within the BTA decomposition region. The additional weight loss of BP‐ZIF‐8‐C1 relative to pristine ZIF‐8 was assigned to the BTA loading content. A calibration curve was first constructed by plotting the known BTA concentrations in mM against the absorbance at 287 nm. For non‐SiO_2_‐coated samples, the sample was first dissolved in 1 M HCl to prepare a 1 mg mL^−1^ stock solution. A defined aliquot of the stock solution was then added to 3 mL of water, and the absorbance of the resulting solution was measured at 287 nm. The measured absorbance was fitted to the calibration curve to determine the BTA concentration. The mass of BTA was calculated using Equation (2):

(2) mBTA=C×V×MBTA

where C (mol L^−1^) is the BTA concentration obtained from the calibration curve, V (L) is the total measured volume, and MBTA is the molecular weight of BTA. The BTA loading was calculated using Equation (3):

(3) $$\text{BTA loading (wt.\%)}=(m_{\mathrm{BTA}}/m_{\text{sample}})\times 100$$

where *m*_sample_ is the mass of sample contained in the measured aliquot. Since the stock solution concentration was 1 mg mL^−1^, the aliquot volume in mL corresponds directly to the sample mass in mg.

### 4.6 Anticorrosion Coating

The zinc‐coated steel plates were sequentially cleaned with ethanol and acetone, then mechanically polished using SiC sandpaper (800–1200 grit) until the zinc coating was removed and a homogeneous steel surface was exposed. The substrates were subsequently dried and treated with UV/ozone to remove residual organic contaminants and enhance surface hydrophilicity, thereby improving adhesion with the WBPU binder. Four coating formulations were prepared: pristine WBPU and WBPU containing 1 wt% ZIF‐8@SiO_2_, 1 wt% BP‐ZIF‐8‐C1@SiO_2_, and 1 wt% BP‐ZIF‐8‐C2@SiO_2_. For each composite coating, the functional filler was added to the WBPU matrix and mechanically stirred at 250 rpm for 30 min until a uniform dispersion was obtained. The coating mixtures were then applied onto cleaned metal plates using a bar coater. The coated substrates were cured at 50 °C for 12 h to form solid coating films. The final dry coating thickness was determined from measurements at multiple positions on each coated substrate.

### 4.7 Electrochemical Measurements

Electrochemical measurements, including potentiodynamic polarization and EIS, were performed to assess the corrosion resistance of the coated plates. All electrochemical measurements were performed using a CHI760E electrochemical workstation in a conventional three‐electrode configuration, employing an Ag/AgCl reference electrode, a platinum counter electrode, and the coated steel plate as the working electrode. EIS measurements were carried out on the coated plates over 39 days of immersion in a 3.5 wt% NaCl solution at room temperature. EIS measurements were conducted using a sinusoidal perturbation of ±10 mV around the open‐circuit potential (OCP) over a frequency range of 100 kHz to 0.01 Hz. The resulting impedance spectra were fitted using an equivalent electrical circuit model provided in the EIS Spectrum Analyzer. Potentiodynamic polarization tests were also performed in 3.5 wt% NaCl solution. Prior to measurement, the electrodes were immersed for 1 h to stabilize the OCP. Polarization was carried out within ±250 mV versus OCP at a scan rate of 0.5 mV s^−1^. The exposed working area of the coated plate was 5.31 cm^2^.

### 4.8 Immersion Test

The coated plates were immersed in a 3.5 wt% NaCl solution at 30 °C, and their anticorrosion performance was evaluated at predetermined time intervals. All immersion tests were performed in a sealed container to minimize evaporation and maintain consistent exposure conditions.

### 4.9 pH‐Dependent Inhibitor Release

To evaluate the pH‐dependent inhibitor release, three aqueous conditions were tested: acidic, neutral, and alkaline conditions. The acidic and alkaline solutions were adjusted to pH 3 and pH 10 using HCl and KOH, respectively, while deionized water with a confirmed pH 7 was used for the neutral condition. In each experiment, 2 mg of sample was dispersed in 2 mL of the corresponding aqueous solution and shaken using a mechanical shaker for 5 s. The sample was then centrifuged, and the supernatant was collected and analyzed by UV–vis spectroscopy at 287 nm. The BTA release percentage was determined by comparing the absorbance of the release supernatant with the maximum absorbance obtained after complete digestion of the sample. For the total BTA measurement, the ZIF‐8/SiO_2_ structure was decomposed using 1 M HCl and 1 M NaOH to release the encapsulated BTA. Prior to UV–vis analysis, the release and total‐digestion solutions were adjusted to the same measurement conditions to minimize matrix‐ and pH‐dependent differences in BTA absorbance. Both the release test and total digestion measurement were prepared at the same sample concentration of 1 mg mL^−1^ and analyzed under the same UV–vis conditions. Therefore, the BTA release percentage was calculated directly from the absorbance ratio using Equation (4) :

(4) $$\text{BTA release (\%)}=[\left(A_{\text{release}}-A_{\text{blank}})/(A_{\text{total}}-A_{\text{blank}}\right)]\times 100$$

where *A*_release_ is the absorbance of the supernatant after release testing, *A*_total_ is the corresponding to the total BTA content after complete sample digestion, and *A*_blank_ is the absorbance of the corresponding blank solution.

## Funding

This work was supported by the Australian Research Council (ARC) through a Linkage Project (LP210200225).

## Conflicts of Interest

The authors declare no conflicts of interest.

## Supporting information

Supplementary Material

## Data Availability

The data that support the findings of this study are available from the corresponding author upon reasonable request.
