# Supplementary material for: Micelle‐Assisted Encapsulation Strategy in Metal‐Organic Framework Nanocontainers Enables Durable Corrosion Protection
Source: Small Sci. 2026 Aug 22;6(8):e70367. doi: 10.1002/smsc.70367 (PMC13516867; doi:10.1002/smsc.70367)
Supplement: Supplementary file 1 [file SMSC-6-e70367-s001.pdf]

Supporting Information

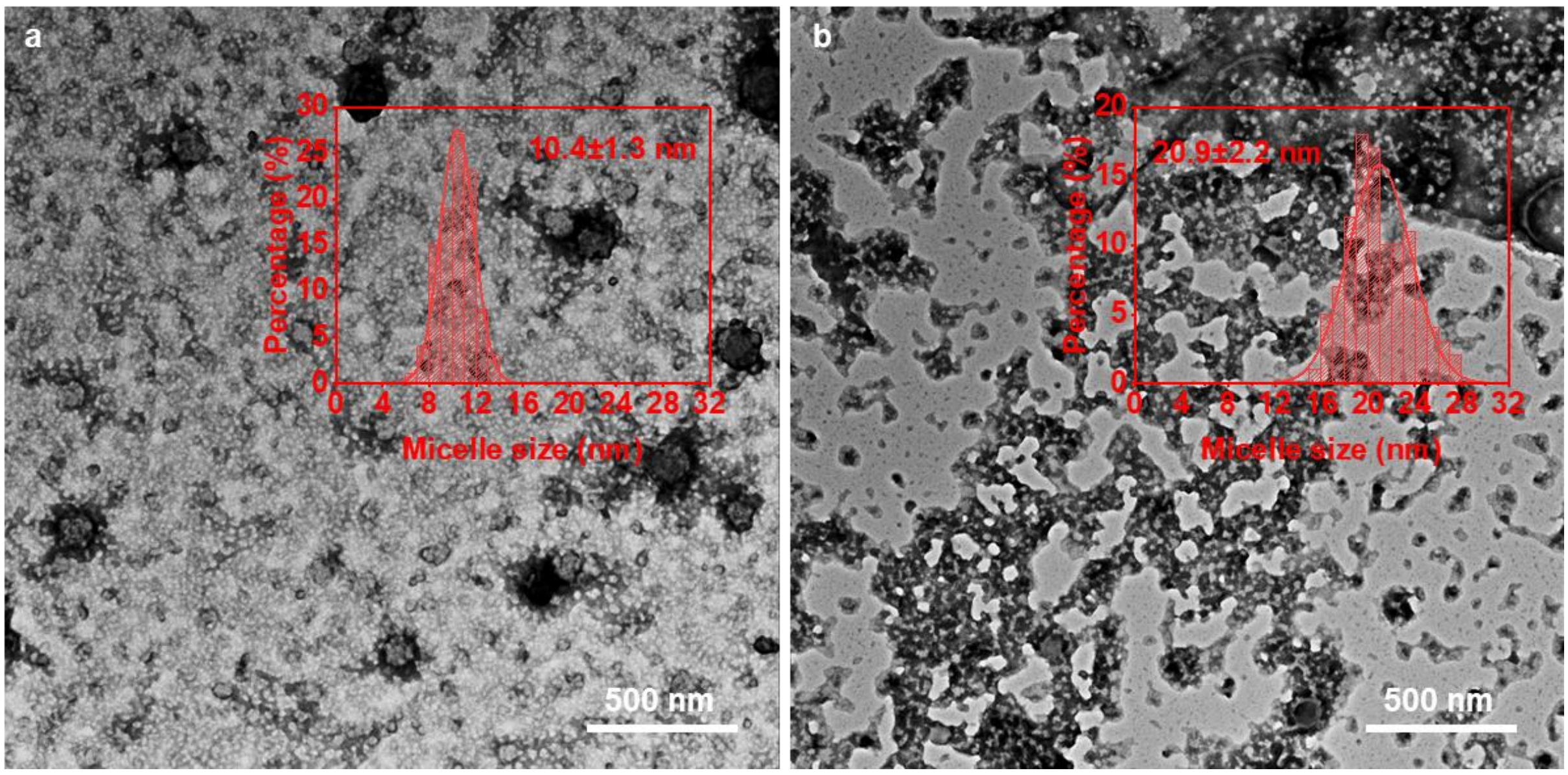

Figure S1. TEM images of P123 micelles (a) without BTA, and (b) with BTA. The polymeric micelles were negatively stained using 1.0 wt% phosphotungstic acid.

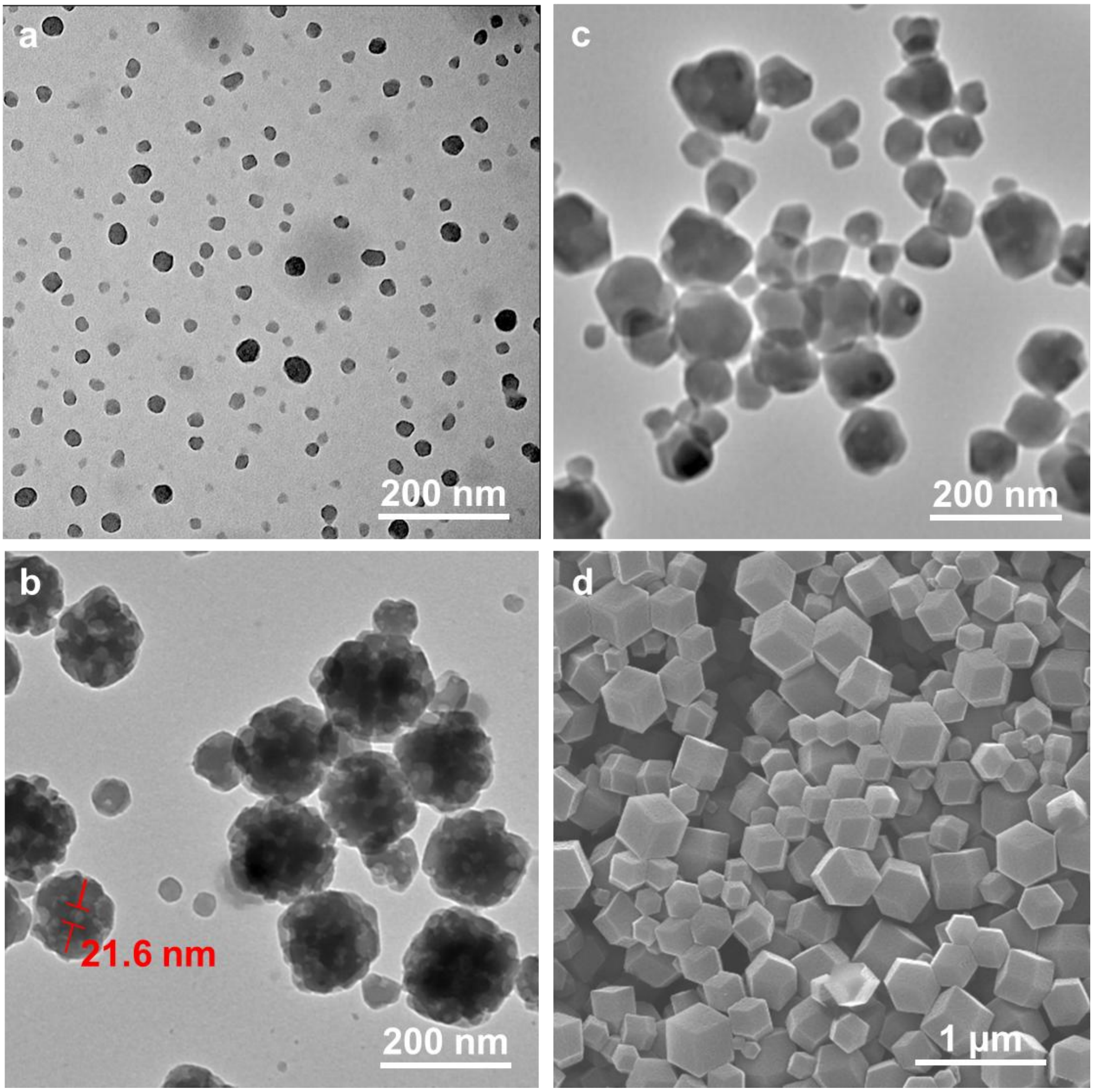

Figure S2. TEM images of samples prepared under different synthesis conditions: (a) in the absence of P123 with BTA, (b) in the presence of P123 followed by micelle removal, and (c) in the presence of P123 without BTA. (d) SEM image of ZIF-8.

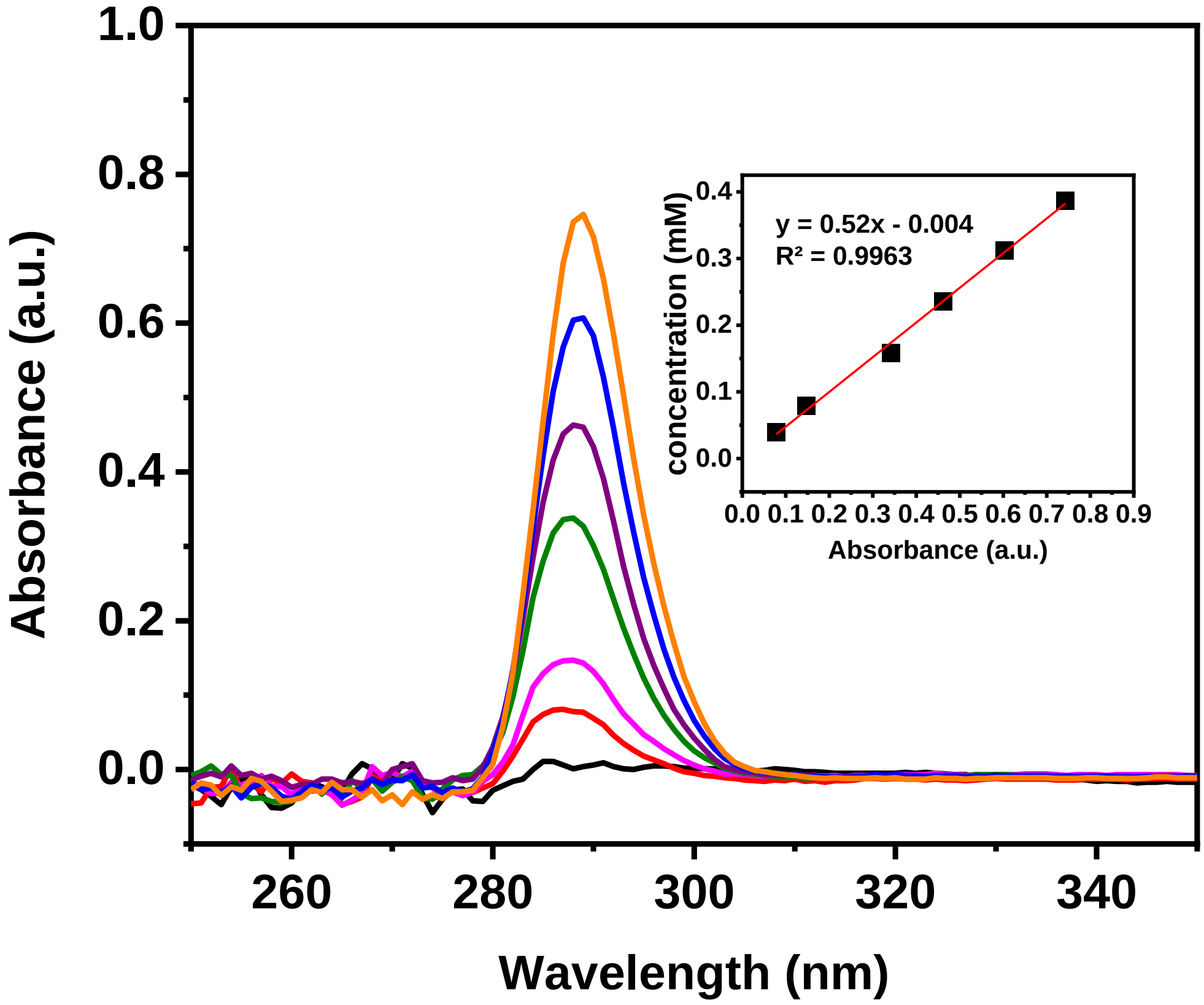

Figure S3. UV–Vis absorption spectra of BTA recorded at different concentrations. The inset shows the linear correlation between the absorbance peak at 287 nm and BTA concentration.

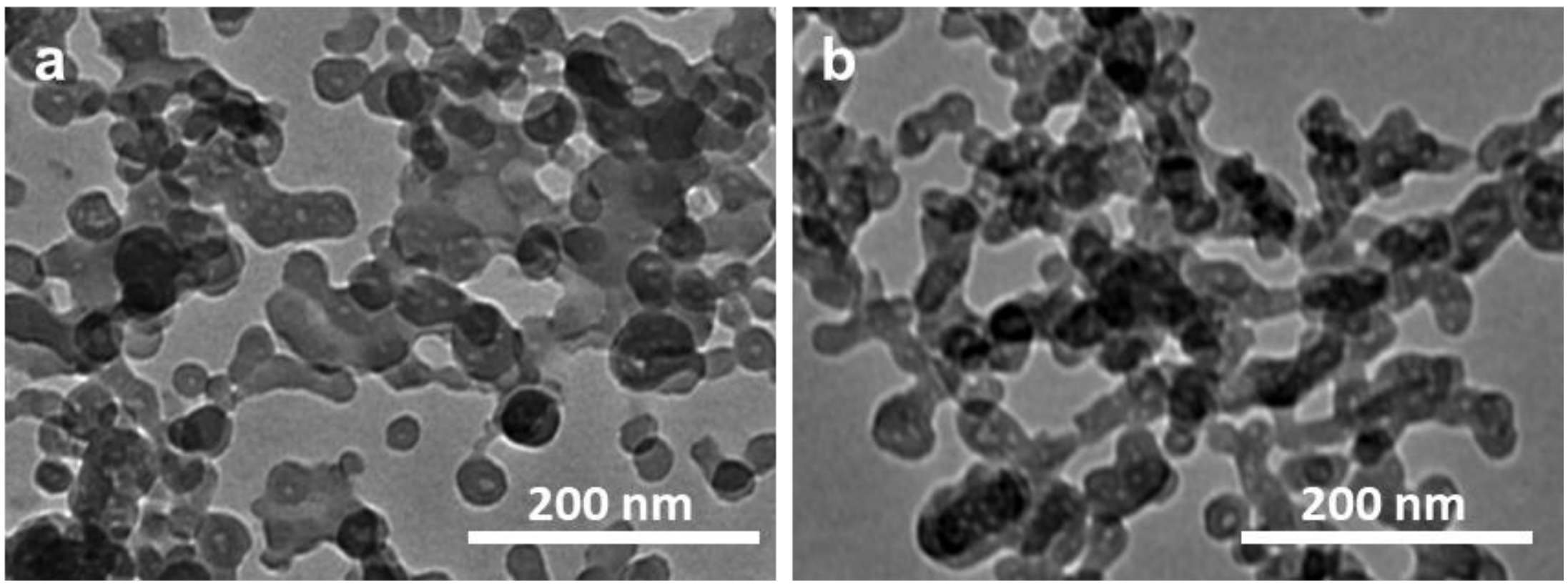

Figure S4. TEM images of BTA/P123-assisted ZIF-8 samples prepared by increasing the BTA amount while maintaining a constant P123 amount, corresponding to BTA/P123 mass ratios of (a) 1.19 and (b) 2.38.

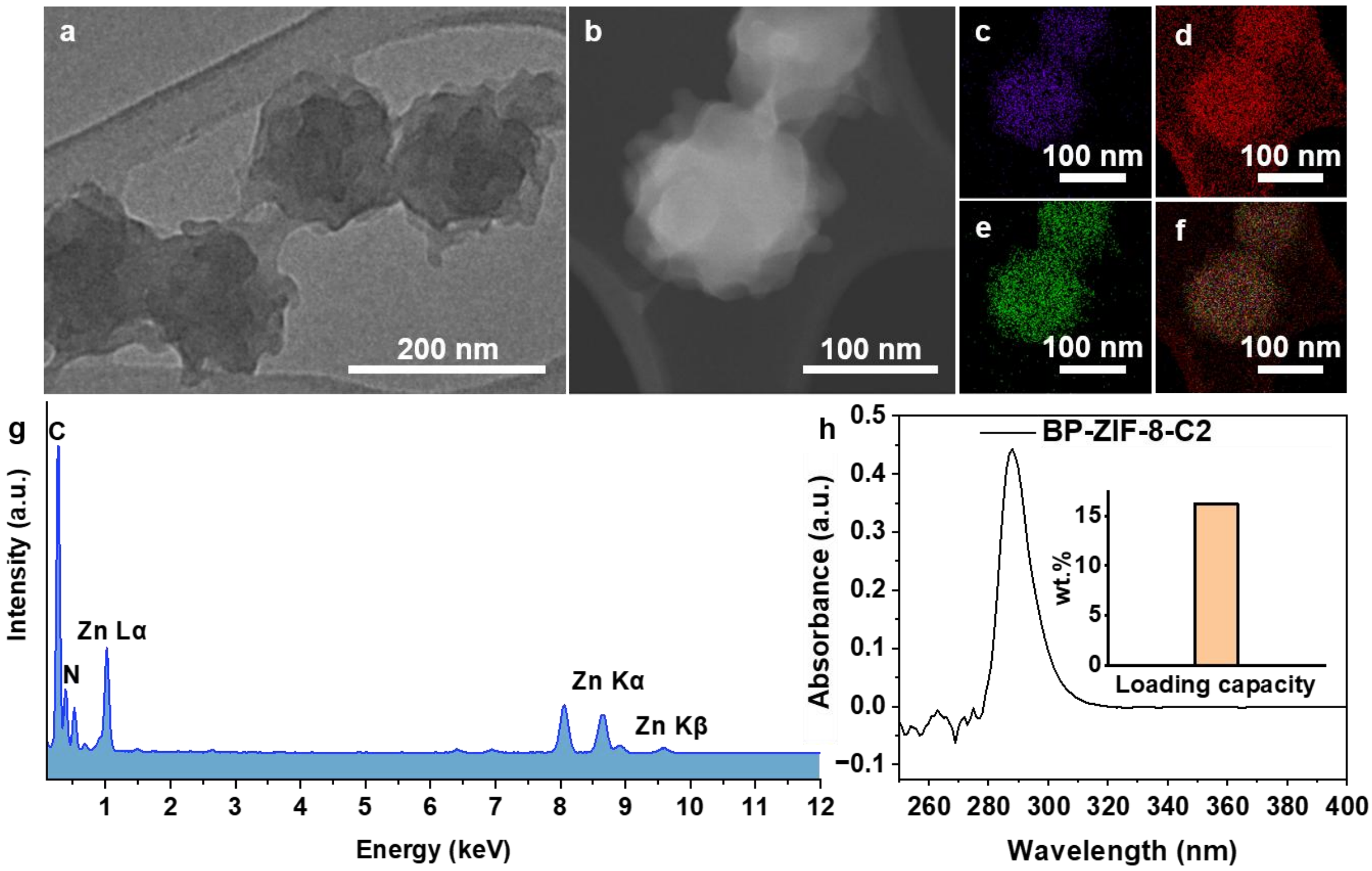

Figure S5. (a) Bright-field and (b) dark-field STEM images of BP-ZIF-8-C2, prepared by increasing both the BTA and P123 amounts to 2.5 times their initial values. The corresponding EDX elemental maps show the spatial distributions of (c) N, (d) C, and (e) Zn, with (f) the overlaid elemental map and (g) the EDX spectrum. (h) UV–Vis absorption spectra used to evaluate BTA loading; the inset shows the corresponding loading capacity. The UV–Vis measurement was performed by first dissolving the sample in 1 M HCl to prepare a 1 mg mL^{-1} stock solution. Then, 600 μL of this stock solution was diluted with 3 mL of water before UV–Vis analysis.

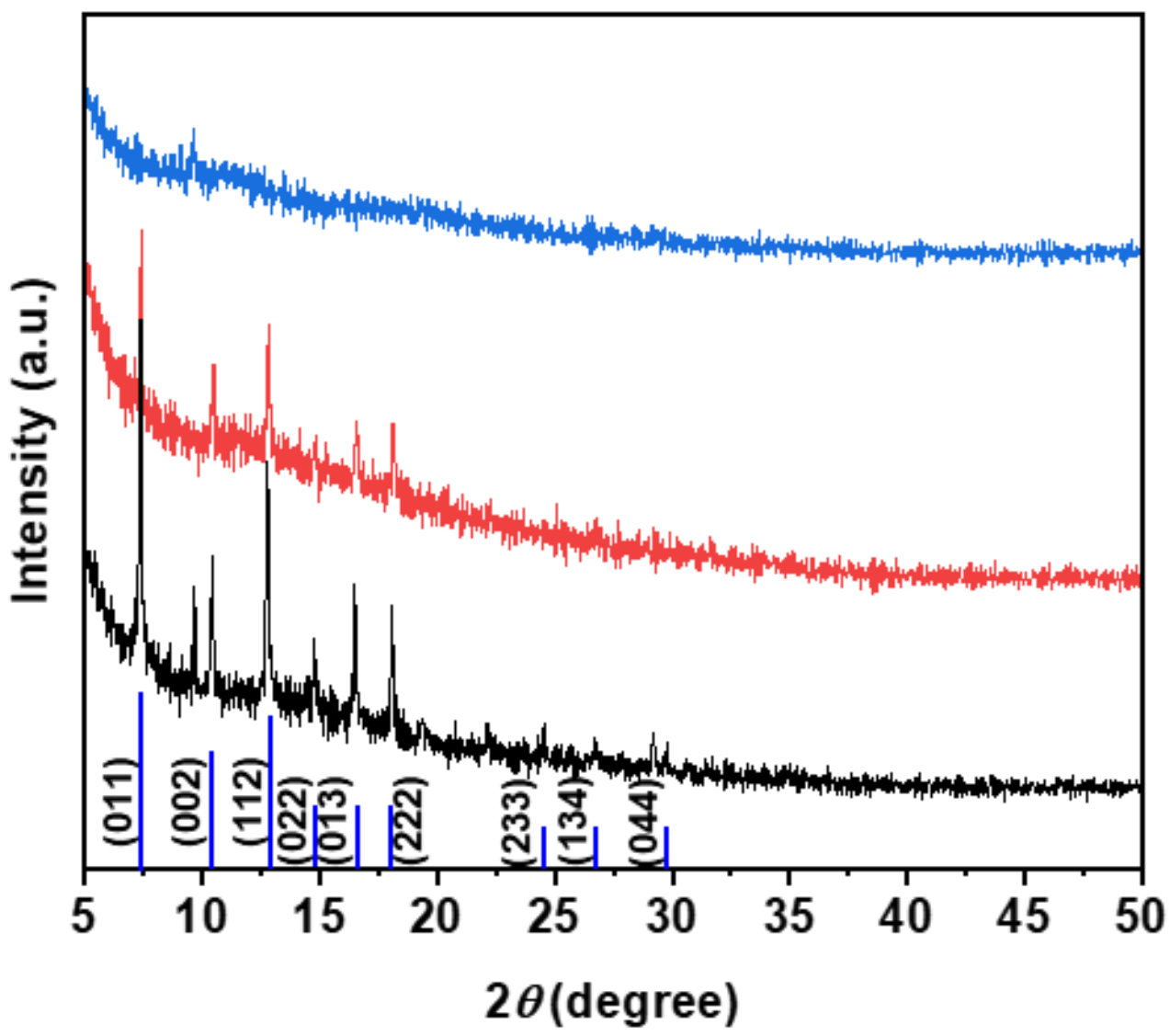

Figure S6. XRD patterns of ZIF-8 samples prepared with different BTA/P123 formulations: BP-ZIF-8-C2 (black line), BP-ZIF-8-R1.19 (red line), and BP-ZIF-8-R2.38 (blue line). The corresponding BTA and P123 amounts and BTA/P123 mass ratios are summarized in Table S1. The simulated ZIF-8 pattern is included as a reference (JCPDS No. 00-062-1030).

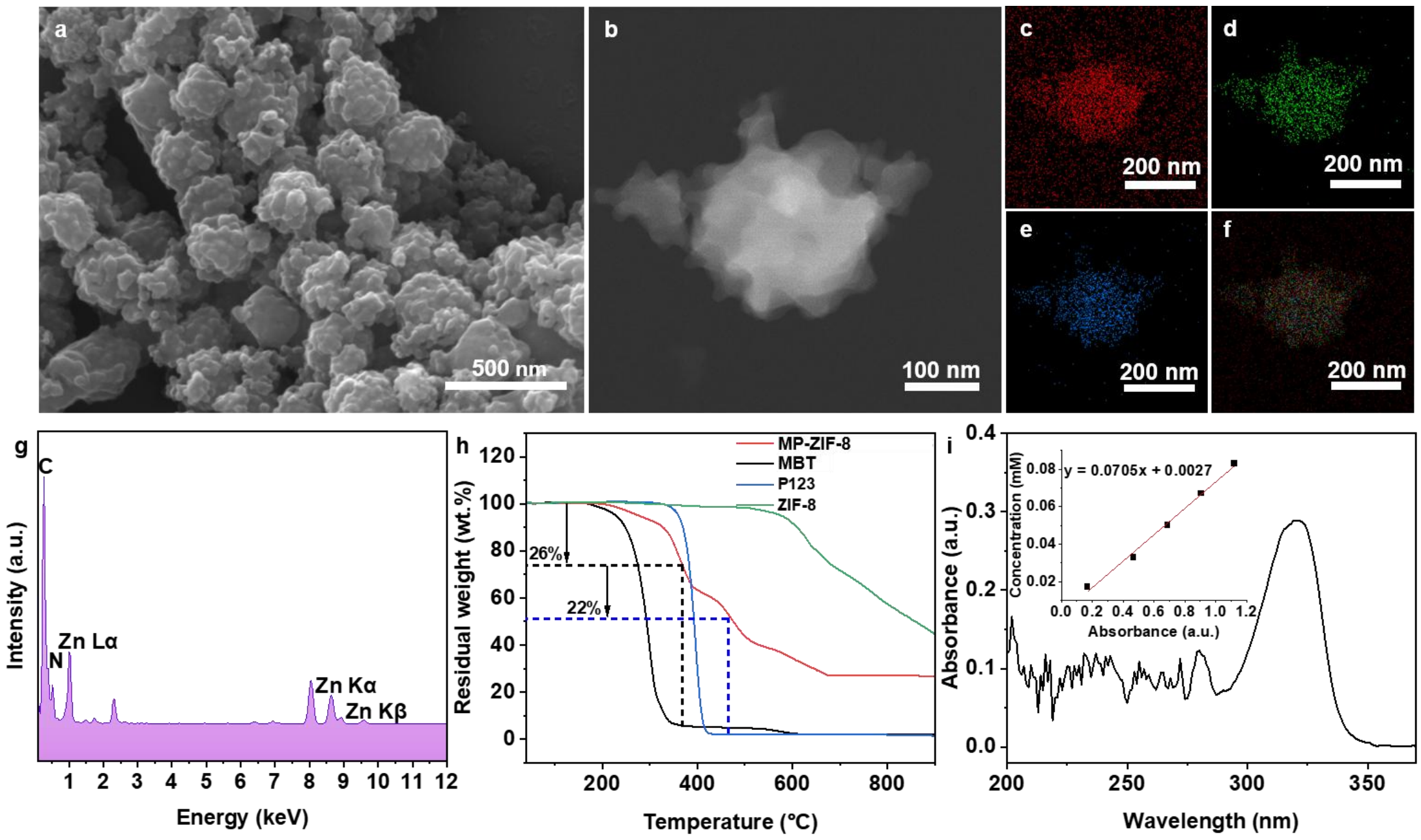

Figure S7. (a) SEM image and (b) HAADF-STEM image of the sample prepared by replacing BTA with MBT, denoted as MP-ZIF-8. The corresponding EDX elemental maps show the spatial distributions of (c) C, (d) Zn, and (e) N, with (f) the overlaid elemental map and (g) the EDX spectrum. (h) TGA curves and (i) UV–Vis absorption spectra of MBT; the inset shows the calibration curve of absorbance versus MBT concentration. The UV–Vis measurement was performed by first dissolving the sample in 1 M HCl to prepare a 1 mg mL^{-1} stock solution. Then, 40 µL of the stock solution was diluted with 3 mL of water before UV–Vis analysis.

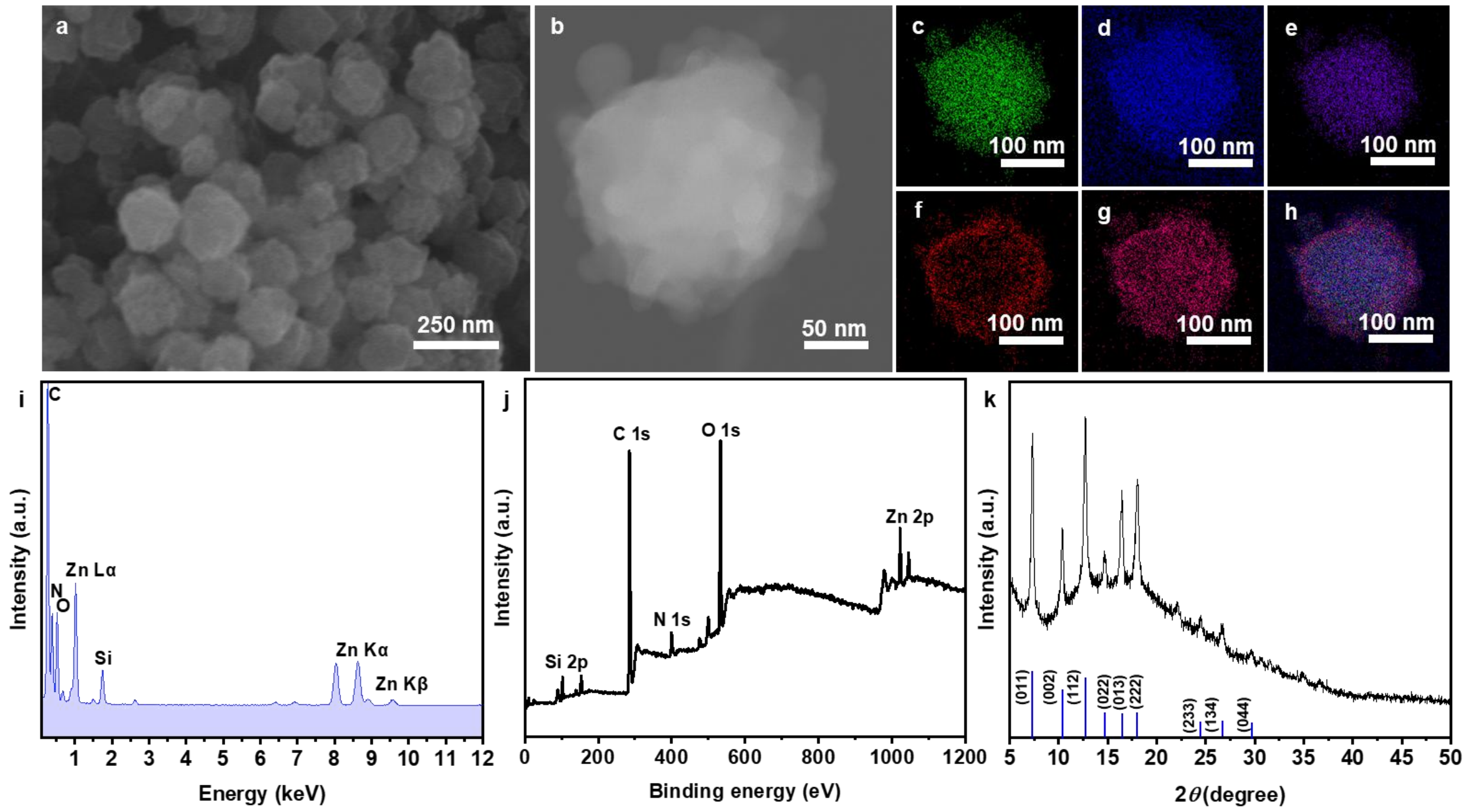

Figure S8. (a) SEM image and (b) HAADF-STEM image of BP-ZIF-8-C1@SiO_2. The corresponding EDX elemental maps show the spatial distributions of (c) Zn, (d) C, (e) N, (f) Si, and (g) O, with (h) the overlaid elemental map and (i) the EDX spectrum. Structural and compositional characterization is further supported by (j) XPS and (k) XRD analyses.

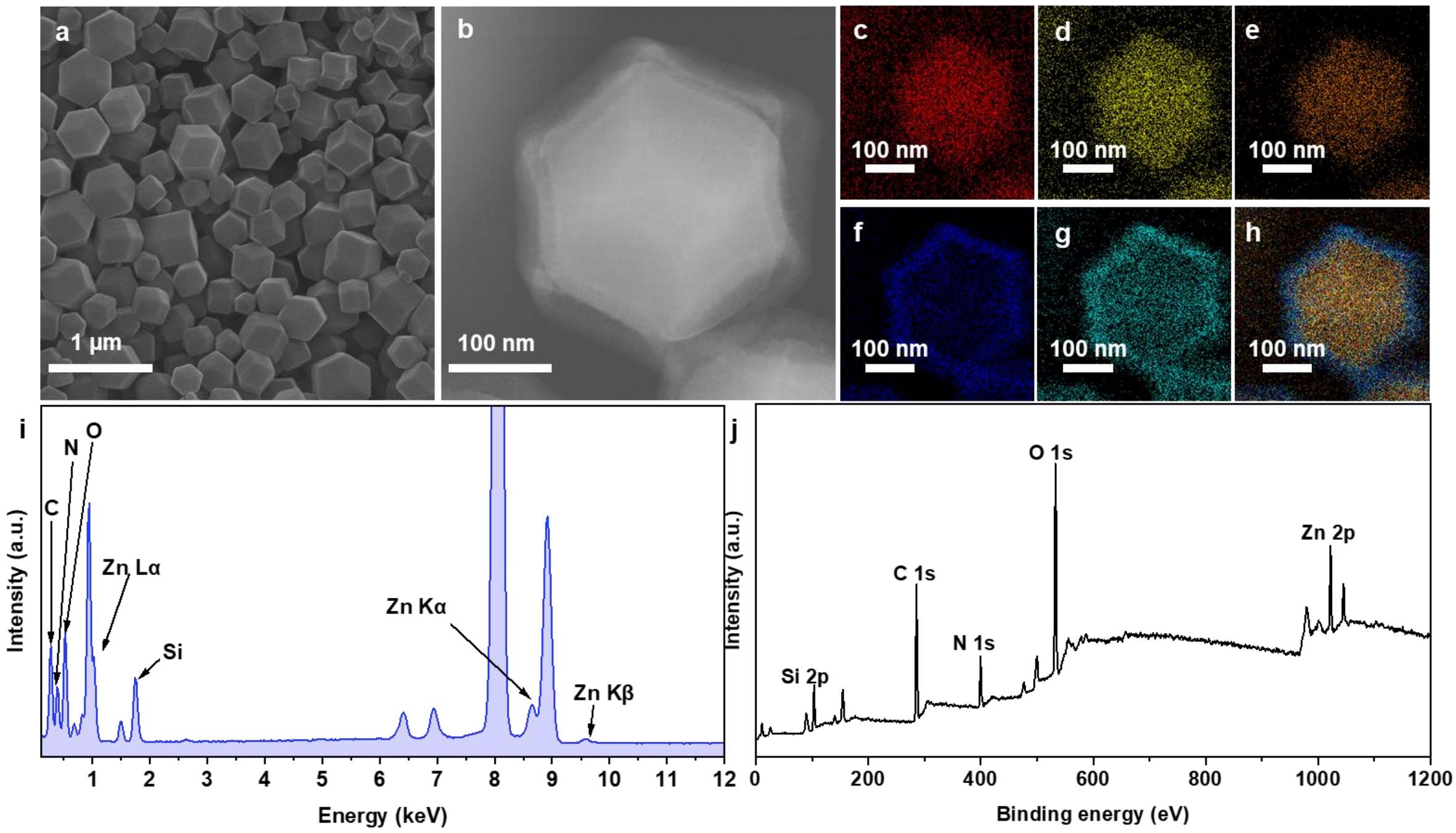

Figure S9. (a) SEM image and (b) HAADF-STEM image of ZIF-8@SiO_2. The corresponding EDX elemental maps show the spatial distributions of (c) C, (d) Zn, (e) N, (f) Si, and (g) O, with (h) the overlaid elemental map and (i) the EDX spectrum. (j) XPS analysis.

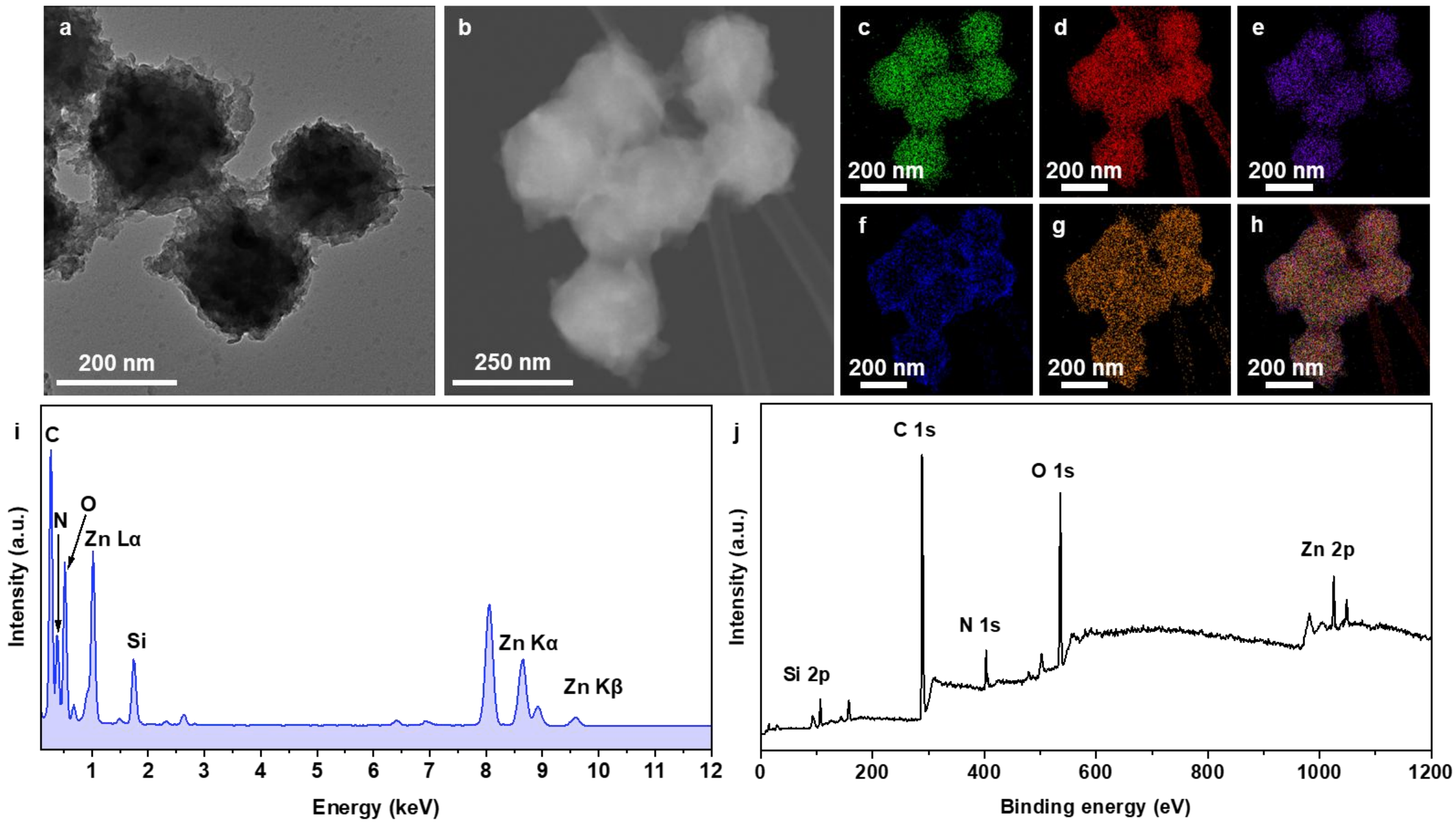

Figure S10. (a) TEM and (b) HAADF-STEM images of BP-ZIF-8-C2@SiO_2, prepared according to the formulation summarized in Table S1. The corresponding STEM–EDX elemental maps show the spatial distributions of (c) Zn, (d) C, (e) N, (f) Si, and (g) O, together with (h) the merged elemental map and (i) the EDX spectrum. (j) XPS spectrum of BP-ZIF-8-C2@SiO_2.

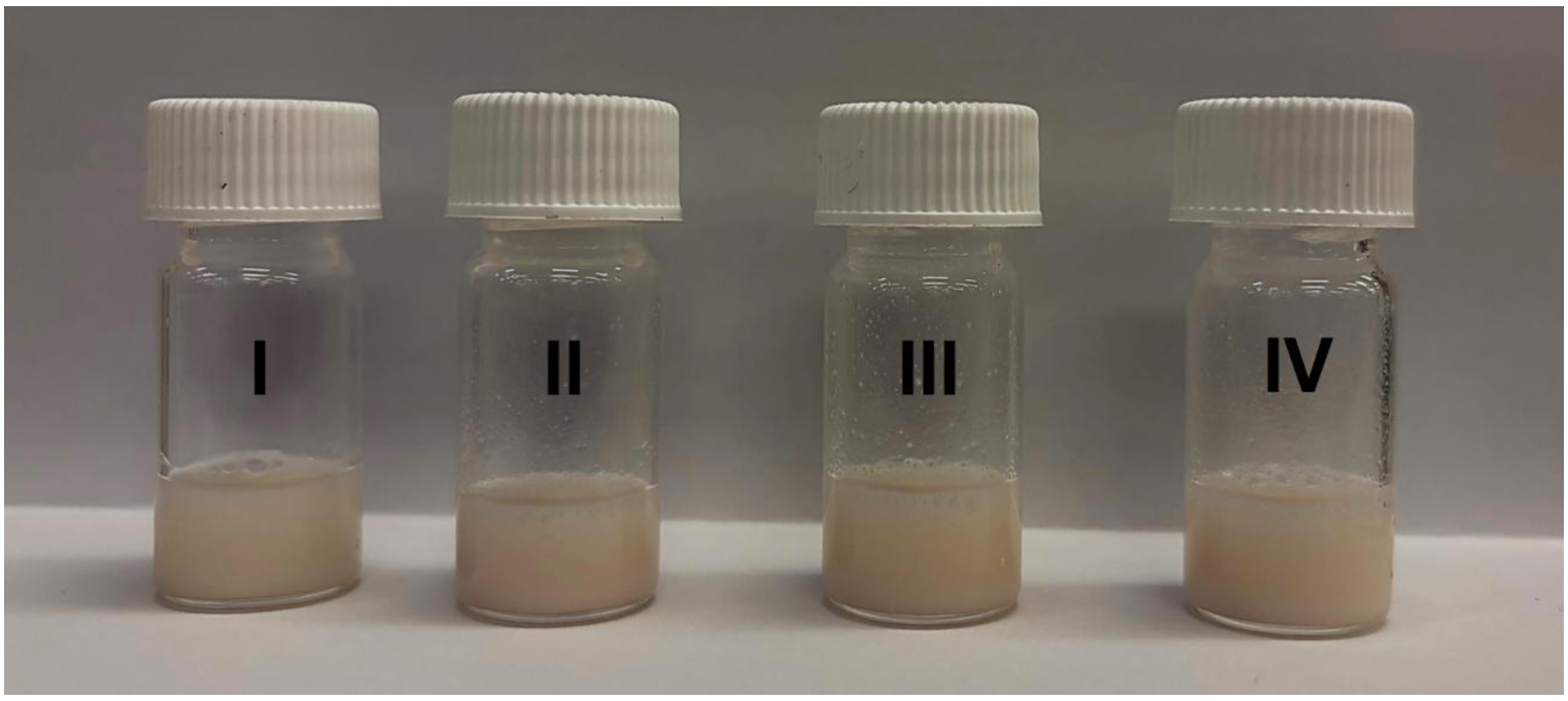

Figure S11. Photograph of the coating suspension after mixing WBPU with different nanocontainer functional fillers: (I) WBPU, (II) WBPU + ZIF-8@SiO_2, (III) WBPU + BP-ZIF-8-C1@SiO_2, and (IV) WBPU + BP-ZIF-8-C2@SiO_2.

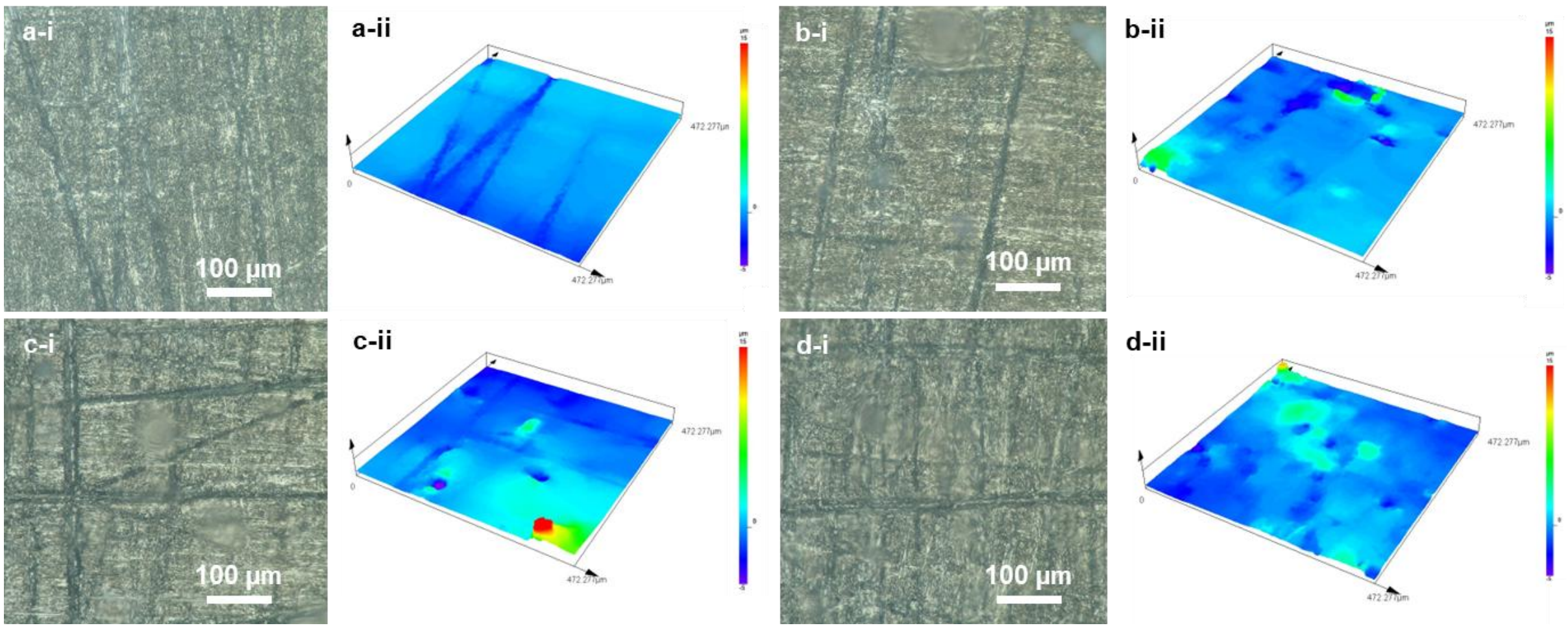

Figure S12. (i) Optical microscopy images and (ii) surface topography images of (a) pristine WBPU, (b) WBPU + ZIF-8@SiO_2, (c) WBPU + BP-ZIF-8-C1@SiO_2, and (d) WBPU + BP-ZIF-8-C2@SiO_2.

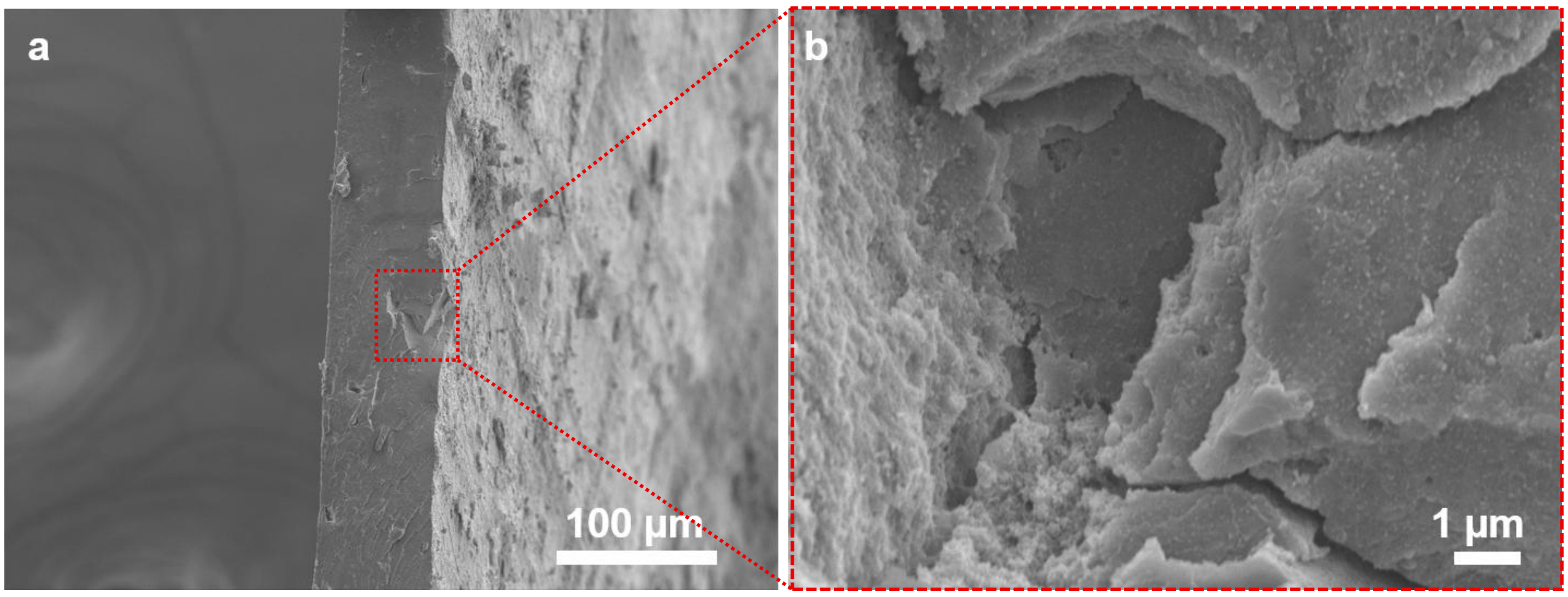

Figure S13. Cross-sectional SEM images of the representative WBPU + BP-ZIF-8-C1@SiO_2 coating at (a) low and (b) high magnification.

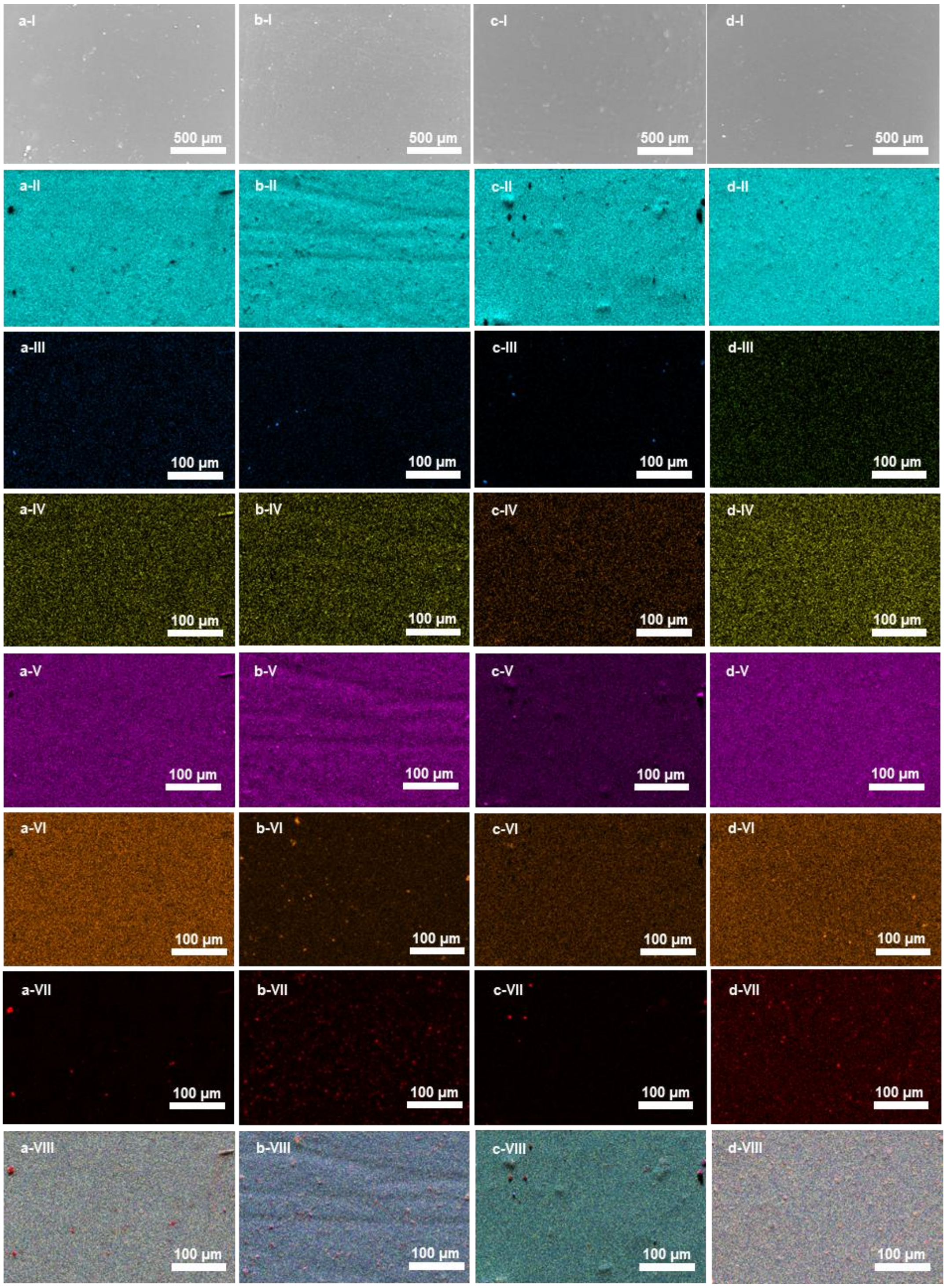

Figure S14. SEM images of coated substrates: (a-I) WBPU, (b-I) WBPU + ZIF-8@SiO_2, (c-I) WBPU + BP-ZIF-8-C1@SiO_2, and (d-I) WBPU + BP-ZIF-8-C2@SiO_2. The corresponding EDX elemental maps are shown in panels (II–VIII), including (II) C, (III) Fe, (IV) N, (V) O, (VI) Si, (VII) Zn, and (VIII) the overlaid elemental map.

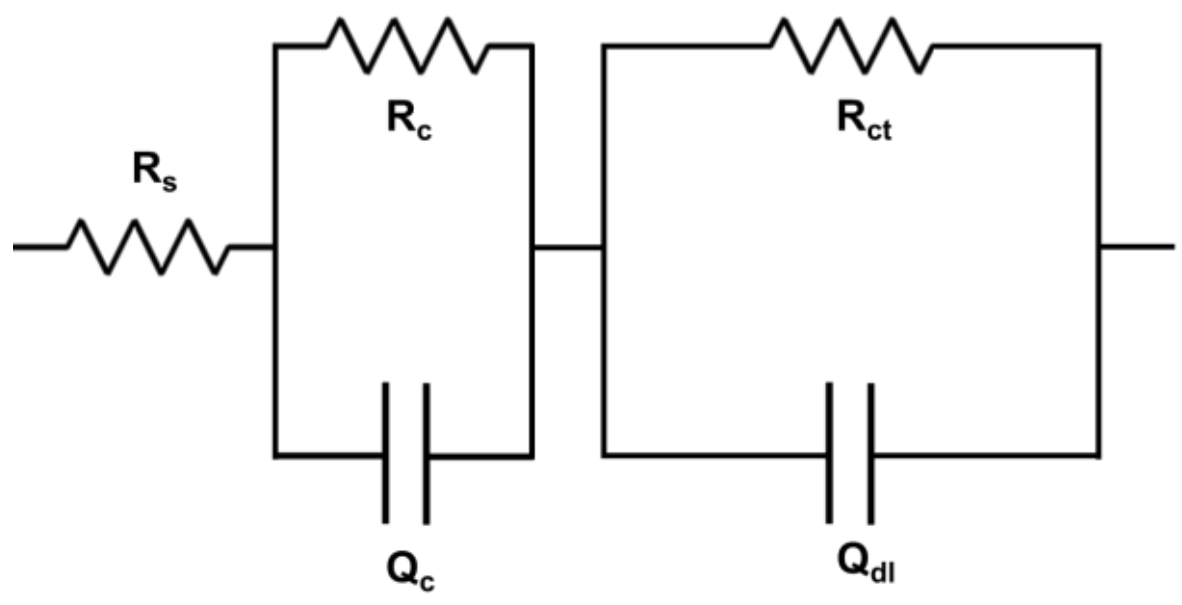

Figure S15. Equivalent circuit model used for EIS fitting.

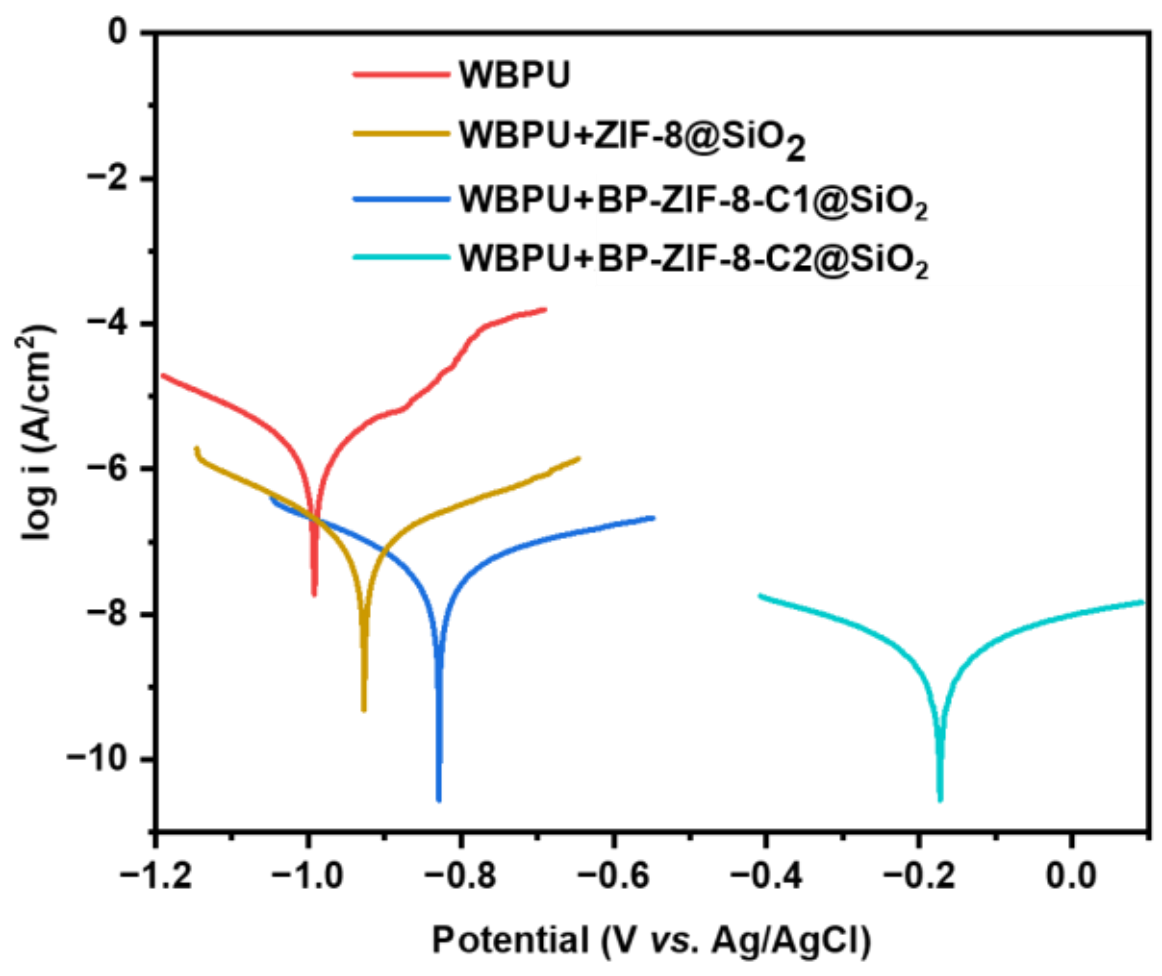

Figure S16. Potentiodynamic polarization measurements of WBPU and WBPU composite coatings incorporating ZIF-8@SiO_2, BP-ZIF-8-C1@SiO_2, and BP-ZIF-8-C2@SiO_2.

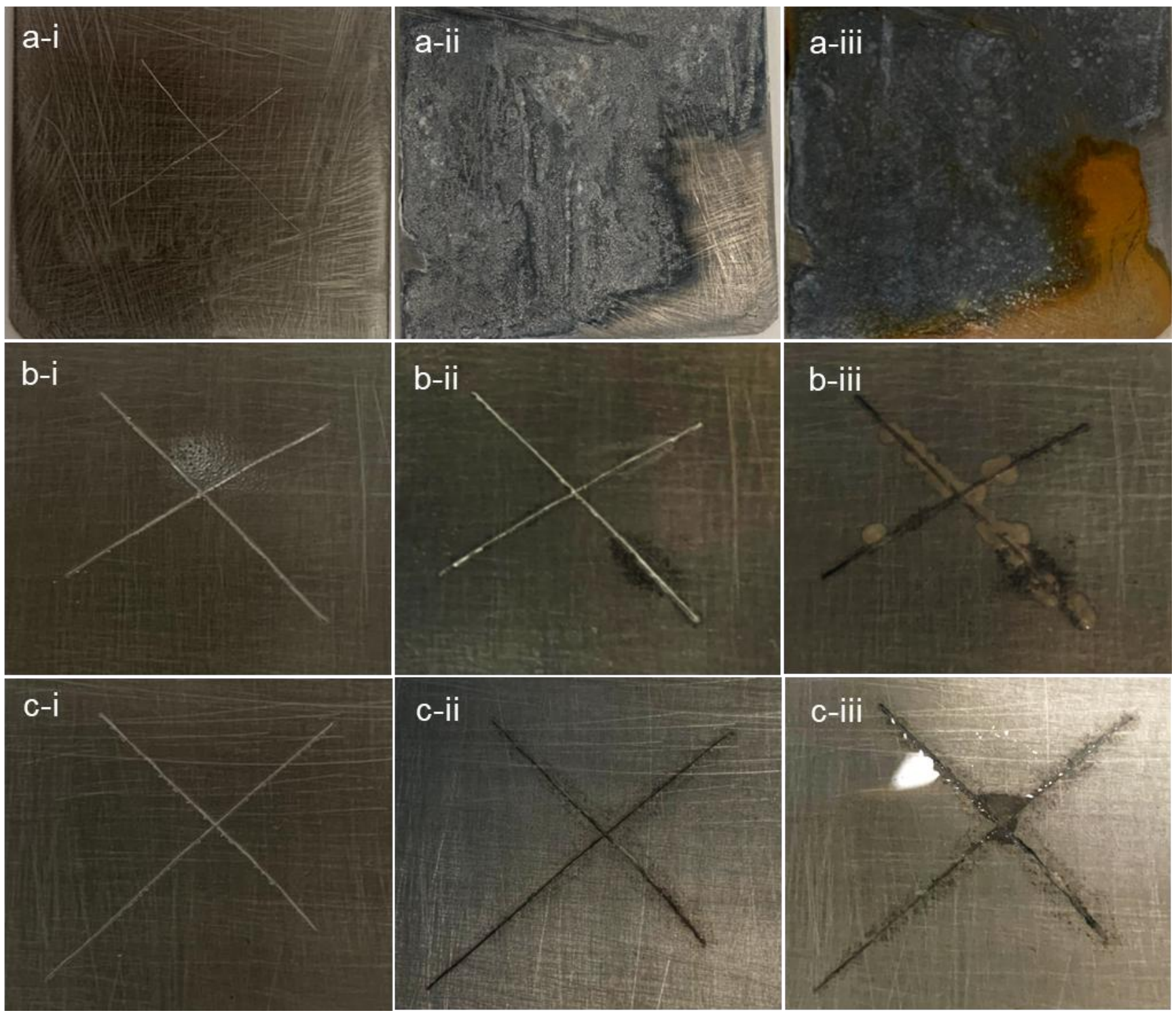

Figure S17. Optical images of (a) bare substrate, (b) WBPU, and (c) WBPU + ZIF-8@SiO_2 coatings with an intentional surface cut after different immersion times: (i) 0 day, (ii) 7 days, and (iii) 14 days.

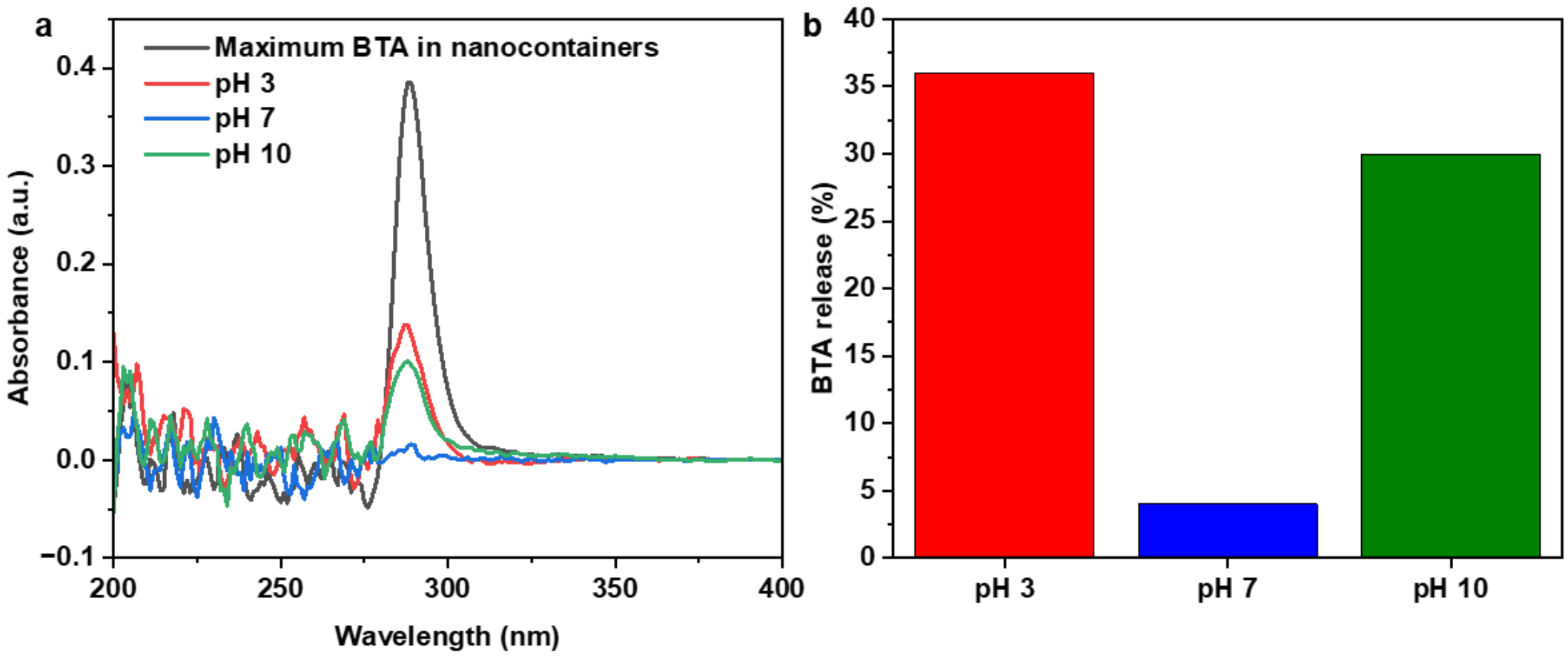

Figure S18. (a) UV–Vis absorption spectra of BTA released from BP-ZIF-8-C2@SiO_2 under varying pH conditions. (b) Corresponding release percentages normalized to the total BTA content determined by complete digestion.

Table S1. Formulation details and nomenclature of BTA/P123-assisted ZIF-8 samples prepared with and without SiO_2 coating.

**Sample name**	**BTA amount (mg)**	**P123 amount (mg)**	**BTA/P123 mass ratio**	**SiO_2 coating**
BP-ZIF-8-C1	119	250	0.48	No
BP-ZIF-8-C2	297.5	625	0.48	No
BP-ZIF-8-R1.19	297.5	250	1.19	No
BP-ZIF-8-R2.38	595	250	2.38	No
BP-ZIF-8-C1@SiO_2	119	250	0.48	Yes
BP-ZIF-8-C2@SiO_2	297.5	625	0.48	Yes

Table S2. Comparative summary of BTA loading capacity, loading mechanism, and process complexity in reported anticorrosion nanocontainers.

Ref.	Material	Inhibitor	Loading capacity	Measurement	Loading method	Loading mechanism	Inhibitor-loading sequence
**1**	BTA@ZNS, 2D Zn(Bim)(OAc) MOF nanosheets	BTA	7.13 wt.%	TGA	Vacuum-assisted BTA loading in methanol	BTA adsorbed onto/within 2D MOF nanosheets	Multi-step
**2**	BTA-SiO_2@ZSM-5 molecular sieve	BTA	9 wt.%	TGA/UV–Vis	Reduced-pressure BTA loading in ethanol	BTA physically adsorbed in SiO_2@ZSM-5 mesopores	Multi-step
**3**	BTA@PDA/PANI microcapsules	BTA	~20 wt.%	TGA and ethanol extraction	Repeated vacuum immersion in BTA/ethanol solution	BTA encapsulated inside PDA/PANI hollow capsules	Multi-step
**4**	Zn-BTA@EHNTs, Zn-BTA complex-plugged etched halloysite nanotubes	BTA	2.8 wt.%	TGA	Vacuum BTA loading into etched HNTs, followed by Zn^{2+} plugging	BTA stored in HNT lumen; Zn-BTA plugs control release	Multi-step
**5**	BNNS/PDA/BTA nanocontainers, denoted as BPB	BTA	16.84 wt.%	UV–Vis	Vacuum-assisted BTA adsorption onto BNNS/PDA	BTA bound through PDA interactions and released by pH changes	Multi-step
**6**	BTA@PHVA/PEI pH-sensitive hybrid hydrogel	BTA	10.12 wt.%	TGA/DSC	One-pot BTA hydrogel formation, followed by PEI wrapping	BTA entrapped in pH-responsive hydrogel network	One-pot nanocontainer synthesis, followed by coating incorporation
**7**	HMSN-BTA@ZIF-8, ZIF-8-capped hollow mesoporous silica	BTA	12.58 wt.%	TGA	Vacuum BTA loading into HMSN, followed	BTA stored in HMSN pores/cavity; ZIF-8 acts as pH gatekeeper	Multi-step

	nanoparticles				by ZIF-8 capping		
**8**	rGO–CD–BTA, reduced graphene oxide/β-cyclodextrin nanocontainers	BTA	24 wt.%	DSC	Reduced-pressure BTA loading into rGO–CD	BTA retained by β-CD host–guest inclusion	Multi-step
**9**	BTA-loaded halloysite clay nanotubes	BTA	4.0 wt.%	UV–Vis	Vacuum-assisted BTA loading into halloysite nanotubes	BTA physically trapped inside halloysite lumen	Multi-step
**10**	HSNs-M/BTA, hollow silica nanocapsules with $Mg(OH)_2$ precipitate in the shell	BTA	28.7 wt.%	TGA	One-step BTA encapsulation during hollow silica formation	In situ BTA encapsulation during inverse-microemulsion formation of hollow silica nanocapsules	One-step nanocapsule synthesis, followed by hydrophobic surface modification and coating incorporation
**This work**	BP-ZIF-8-C2 nanocontainers	BTA	16.2 wt.%	UV–Vis	P123-assisted in situ BTA encapsulation during ZIF-8 growth	Micelle-assisted BTA confinement in ZIF-8	Integrated nanocontainer formation and inhibitor encapsulation

Reported loading capacities were taken from the literature and provide only an approximate comparison because measurement methods differ among studies.

Table S3. Average dry thicknesses of the prepared WBPU-based coatings.

**Sample name**	**Thickness (μm)**
WBPU	58.66 ± 7.40
WBPU + ZIF-8@SiO_2	62.88 ± 3.40
WBPU + BP-ZIF-8-C1@SiO_2	64.90 ± 2.93
WBPU + BP-ZIF-8-C2@SiO_2	65.52 ± 3.64

Table S4. EIS fitting parameters of WBPU-based coatings with different compositions obtained using the proposed equivalent circuit model.

**Film**	**Days**	**R_s ($\Omega\cdot cm^2$)**	**R_c ($\Omega\cdot cm^2$)**	**Q_c ($S\cdot s^{n1}\cdot cm^{-2}$)**	**n_1**	**R_{ct} ($\Omega\cdot cm^2$)**	**Q_{dl} ($S\cdot s^{n2}\cdot cm^{-2}$)**	**n_2**
WBPU	1	920	421000	5.86×10^{-6}	0.71	237000	1.05×10^{-7}	0.56
WBPU	14	531	47700	2.58×10^{-5}	0.59	1620	2.93×10^{-8}	0.83
WBPU	20	425	53800	3.62×10^{-5}	0.71	308	2.69×10^{-7}	0.88
WBPU	28	266	375	1.13×10^{-7}	0.99	104000	3.36×10^{-5}	0.74
WBPU	39	148	14000	6.07×10^{-5}	0.94	36900	9.42×10^{-5}	0.69
WBPU+ZIF-8@SiO_2	1	789	604000	6.34×10^{-6}	0.72	114000	1.38×10^{-8}	0.78
WBPU+ZIF-8@SiO_2	14	621	227000	1.30×10^{-5}	0.74	3920	3.13×10^{-8}	0.81
WBPU+ZIF-8@SiO_2	20	531	233000	1.32×10^{-5}	0.73	3860	5.84×10^{-8}	0.77
WBPU+ZIF-8@SiO_2	28	1590	297000	1.27×10^{-5}	0.71	2680	9.18×10^{-5}	0.77
WBPU+ZIF-8@SiO_2	39	531	306000	1.03×10^{-5}	0.67	2490	4.73×10^{-8}	0.81
WBPU+BP-ZIF-8-C1@SiO_2	1	994	346000	8.67×10^{-6}	0.68	535000	3.23×10^{-8}	0.67
WBPU+BP-ZIF-8-C1@SiO_2	14	1050	585000	1.24×10^{-5}	0.81	589000	4.07×10^{-8}	0.64
WBPU+BP-ZIF-8-C1@SiO_2	20	1060	481000	3.80×10^{-8}	0.66	733000	8.19×10^{-6}	0.63
WBPU+BP-ZIF-8-C1@SiO_2	28	1060	282000	4.52×10^{-8}	0.65	869000	7.15×10^{-6}	0.64
WBPU+BP-ZIF-8-C1@SiO_2	39	1590	221000	4.68×10^{-8}	0.65	1310000	7.34×10^{-6}	0.63
WBPU+BP-ZIF-8-	1	1170	5180000	5.91×10^{-9}	0.71	1680000	1.82×10^{-6}	0.43

C2@SiO_2								
WBPU+BP-ZIF-8-C2@SiO_2	14	1840	1610000	7.40×10^{-7}	0.40	5310000	5.61×10^{-9}	0.74
WBPU+BP-ZIF-8-C2@SiO_2	20	1090	5980000	6.30×10^{-9}	0.70	1590000	3.29×10^{-6}	0.64
WBPU+BP-ZIF-8-C2@SiO_2	28	1090	9550000	6.30×10^{-9}	0.70	1590000	3.29×10^{-6}	0.72
WBPU+BP-ZIF-8-C2@SiO_2	39	1090	12600000	6.30×10^{-9}	0.69	2660000	3.29×10^{-6}	0.72

R_s denotes the solution resistance; R_c the coating resistance; Q_c the constant phase element parameters associated with the coating; and Rct the charge-transfer resistance at the metal/coating interface; Q_{dl} represents the constant phase element associated with the electrical double layer at the metal/coating interface.

Table S5. Electrochemical parameters obtained from potentiodynamic polarization measurements of the coatings after 39 days of immersion testing.

**Sample**	E_{corr} **(V vs. Ag/AgCl)**	i_{corr} **(A cm^{-2})**	**Corrosion rates *(mm/year)***
WBPU	-0.992	2.08×10^{-6}	0.0242
WBPU + ZIF-8@SiO_2	-0.926	9.87×10^{-8}	0.00115
WBPU+ BP-ZIF-8-C1@SiO_2	-0.823	3.58×10^{-8}	0.000417
WBPU+ BP-ZIF-8-C2@SiO_2	-0.173	3.2×10^{-9}	0.0000372

References

1. Zhang, X., Zhu, G., Kang, F., Zhang, G., Tan, Q., Wu, M., Tan, Y. & Liu, F.-Q. Fabrication of epoxy composite coating with superior anticorrosive properties using a pH-responsive system based on 2D Zn-MOF nanosheets. *Colloids Surf. A Physicochem. Eng. Asp.* 703, 135285 (2024).
2. Zhang, Y.-F., Hu, C., Zhao, Z.-B., Ma, Y., Lin, D.-D., Du, X.-Y., Liu, J.-D. & Li, W.-L. Fabrication of BTA-loaded mesoporous silica-encapsulated ZSM-5 molecular sieve for enhancing anti-corrosion performance of waterborne epoxy coatings. *Prog. Org. Coat.* 172, 107160 (2022).
3. Feng, Y., Li, H., Zhang, M., Jin, J., Zhang, B., Wang, Y. & Li, Z. Preparation of BTA@PDA/PANI microcapsules and anti-corrosion performance of self-healing epoxy coatings on low carbon steel. *Colloids Surf. A Physicochem. Eng. Asp.* 649, 129481 (2022).
4. Meng, X., Jing, M., Xu, X., Su, Y. & Tang, Y. Zn-BTA complex-plugged halloysite nanocontainers for construction of stimuli-responsive epoxy coating. *Prog. Org. Coat.* 196, 108727 (2024).
5. Wang, L., Zhang, J., Liu, T., Sun, F., Wang, Q., Wang, Y., Zhang, T., Xu, J. & Fu, J. An active self-healing and UV-resistant anticorrosion coating based on loading-enhanced nanocontainers tailored for Q235 carbon steel protection. *Prog. Org. Coat.* 201, 109128 (2025).
6. Wen, J., Lei, J., Chen, J., Gou, J., Li, Y. & Li, L. An intelligent coating based on pH-sensitive hybrid hydrogel for corrosion protection of mild steel. *Chem. Eng. J.* 392, 123742 (2020).
7. Zhou, C., Li, Z., Li, J., Yuan, T., Chen, B., Ma, X., Jiang, D., Luo, X., Chen, D. & Liu, Y. Epoxy composite coating with excellent anticorrosion and self-healing performances based on multifunctional zeolitic imidazolate framework derived nanocontainers. *Chem. Eng. J.* 385, 123835 (2020).
8. Liu, C., Zhao, H., Hou, P., Qian, B., Wang, X., Guo, C. & Wang, L. Efficient graphene/cyclodextrin-based nanocontainer: synthesis and host–guest inclusion for self-healing anticorrosion application. *ACS Appl. Mater. Interfaces* 10, 36229–36239 (2018).
9. Abdullayev, E., Abbasov, V., Tursunbayeva, A., Portnov, V., Ibrahimov, H., Mukhtarova, G. & Lvov, Y. Self-healing coatings based on halloysite clay polymer composites for protection of copper alloys. *ACS Appl. Mater. Interfaces* 5, 4464–4471 (2013).
10. Zhao, D., Liu, D. & Hu, Z. A smart anticorrosion coating based on hollow silica nanocapsules with inorganic salt in shells. *J. Coat. Technol. Res.* 14, 85–94 (2017).
